# “Two Birds with One Stone” Ruthenium(II) Complex Probe for Biothiols Discrimination and Detection In Vitro and In Vivo

**DOI:** 10.1002/advs.202000458

**Published:** 2020-05-29

**Authors:** Chaolong Liu, Jianping Liu, Wenzhu Zhang, Yong‐Lei Wang, Qi Liu, Bo Song, Jingli Yuan, Run Zhang

**Affiliations:** ^1^ State Key Laboratory of Fine Chemicals School of Chemical Engineering Dalian University of Technology Dalian 116024 China; ^2^ Australian Institute for Bioengineering and Nanotechnology The University of Queensland, St. Lucia Brisbane QLD 4072 Australia; ^3^ Department of Materials and Environmental Chemistry Stockholm University Stockholm SE‐10691 Sweden

**Keywords:** biothiols, ruthenium complexes, time‐gated luminescence, two birds with one stone probes

## Abstract

In this work, a “two birds with one stone” ruthenium(II) complex probe, Ru‐NBD, is proposed as an effective tool for biothiols detection and discrimination in vitro and in vivo. Ru‐NBD is nonluminescent due to the quenching of Ru(II) complex emission by photoinduced electron transfer (PET) from Ru(II) center to NBD and the quenching of NBD emission through 4‐substitution with “O” ether bond. Ru‐NBD is capable of reacting with Cys/Hcy to form long‐lived red‐emitting Ru‐OH and short‐lived green‐emitting NBD‐NR, while reacting with GSH to produce Ru‐OH and nonemissive NBD‐SR. The long lifetime emission of Ru(II) complex allows elimination of short lifetime background and NBD‐NR fluorescence for total biothiols detection (“bird” one) by time‐gated luminescence (TGL) analysis, and the remarkable difference in luminescence color response allows discrimination GSH and Cys/Hcy (“bird” two) through steady‐state luminescence analysis. Ru‐NBD features high sensitivity and selectivity, rapid luminescence response, and low cytotoxicity, which enables it to be used as the probe for luminescence and background‐free TGL detection and visualization of biothiols in live cells, zebrafish, and mice. The successful development of this probe is anticipated to contribute to the future biological studies of biothiols roles in various diseases.

## Graphical abstract

A “two birds with one stone” ruthenium(II) complex probe, Ru‐NBD, is reported for biothiols detection and discrimination in vitro and in vivo. Ru‐NBD (“stone”) is capable of detecting total biothiols (“bird” one) by time‐gated luminescence (TGL) analysis and discriminating glutathione and cysteine/homocysteine (“bird” two) through steady‐state luminescence analysis.

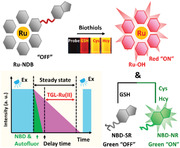

## 1 Introduction

Advances of biomedical and (pre)clinical research heavily rely on the innovation of new techniques for the analysis of biomolecules in vitro and in vivo. The demands for new technologies provide huge space for the development of various bioanalytical methods to probe dynamics of specific biomolecules (biomarkers) at the cellular/molecular level.^[^ ^1^ ^]^ Of various methods, luminescent bioassay has launched biological studies into a new realm for a better understanding the dynamics of biomarkers in live single cells and animals.^[^ ^2^ ^]^ In particular, responsive luminescence probes that can rapidly and effectively detect and discriminate various analytes, such as biothiols, have attracted increasingly attention in the past few years.^[^ ^3^ ^]^

Biothiols, including glutathione (GSH), cysteine (Cys), and homocysteine (Hcy), play critical roles in maintaining intracellular redox activities in biological systems.^[^ ^4^ ^]^ Abnormal concentrations of biothiols are associated with various diseases such as Parkinson's disease, Alzheimer's disease, cardiovascular disease, cancer etc.^[^ ^5^ ^]^ Specifically, alterations of normal intracellular GSH level are implicated in liver damage, leucocyte loss, psoriasis, cancer, HIV infection, etc.^[^ ^6^ ^]^ The deficiency of Cys is closely associated with slow growth, edema, lethargy, liver damage, loss of muscle and fat, skin lesions etc.^[^ ^7^ ^]^ The abnormal Hcy level is able to cause or exacerbate cardiovascular and Alzheimer's diseases.^[^ ^8^ ^]^ In this context, sensitive detection of total biothiols (“bird” one) and selective discrimination each of these biothiols (“bird” two) are of great importance for investigating their correlation with a particular subset of disease states. For the detection of biothiols, most of the responsive probes have been developed either for total biothiols or for one specific biothiol determination, that is, two different probes are required to detect and discriminate biothiols separately. Therefore, it is highly demanded to develop new “two birds with one stone” probe that can simultaneously detect total biothiols and discriminate each of them in live cells and animals,^[^ ^9^ ^]^ and this probe could be obtained through rational engineering and evaluation of luminescent ruthenium(II) complex probes.

As one of most important luminophore, luminescent Ru(II) complexes are increasingly contributing to the field of bioassay and bioimaging due to their abundant properties in optical physics, chemistry, and electrochemistry.^[^ ^10^ ^]^ In the last few years, a variety of responsive Ru(II) complex probes have been developed for the detection of proteins, small biomolecules, and ions.^[^ ^11^ ^]^ Through exploring the mechanisms of photo‐induced electron transfer (PET),^[^ ^12^ ^]^ intramolecular charge transfer (ICT)^[^ ^13^ ^]^ and luminescence resonance energy transfer (LRET),^[^ ^14^ ^]^ our previous research has also contributed to the development of responsive Ru(II) complex probes for biomarkers detection and imaging in live cells and organisms. These luminescent probes are generally designed by integrating a biomolecule responsive moiety with Ru(II) luminophore, thus the luminescence signals can be rationally modulated for bioassay. The long‐lived emission of Ru(II) complex endows these probes in time‐gated luminescence (TGL) bioassay.^[^ ^15^ ^]^ Moreover, some of responsive Ru(II) complex probes have recently been reported for biothiols detection,^[^ ^13^, ^16^ ^]^ while the one that can discriminate three biothiols is scarce. In particular, none of Ru(II) complex probes is currently available for simultaneous detection and discrimination of biothiols in vitro and in vivo.

**Scheme 1:**
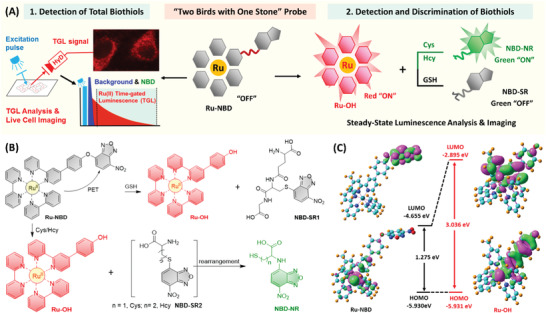
“Two birds with one stone” Ru(II) complex responsive probe for biotihols detection and discrimination. A) Schematic representation of the strategy for the detection of total biothiols (“bird” one) and discrimination of GSH and Cys/Hcy (“bird” two) using Ru‐NBD probe (“stone”). B) Schematic illustration of the molecular structure of Ru‐NBD probe and the response mechanism of Ru‐NBD to GSH and Cys/Hcy. C) Molecular orbitals (MOs) of Ru‐NBD and Ru‐OH based on emission transitions theoretical computation.

In this work, a responsive Ru(II) complex probe (“stone”), Ru‐NBD, is presented for the detection of total biothiols (“bird” one) and discrimination of GSH and Cys/Hcy (“bird” two) (**Scheme** **1**). The probe Ru‐NBD is developed by conjugating two different luminophore, 7‐nitro‐2,1,3‐benzoxadiazoles (NBD) and Ru(II)‐bipyridine complex through a responsive “O” ether bond. Although dual luminophore, Ru‐NBD is expected to be weakly luminescent due to the sophisticated design strategy: i) quenching of Ru(II) luminescence by PET from Ru(II) center to electron withdrawing NBD moiety; ii) quenching of NBD emission by 4‐substitute with “O” ether bond. Biothiols triggered nucleophilic reaction leads to liberation of long lifetime red‐emitting Ru‐OH, short lifetime green‐emitting NBD‐NR (R = Cys, Hcy), or nonemissive NBD‐SR (R = GSH), which allows the detection of total biothiols and discrimination of GSH and Cys/Hcy in vitro and in vivo through TGL and steady‐state luminescence analysis, respectively.

## 2 Results and Discussion

### 2.1 Design, Synthesis, and Characterization of Ru‐NBD

Although a variety of luminescence probes have been reported for biothiols detection,^[^ ^17^ ^]^ the one that enables simultaneous detection and discrimination biothiols in a specific biological sample has not been achieved. Taking advantages of Ru(II) complex luminophore, this work reports the “two birds with one stone” strategy for the design and preparation of a new Ru(II) complex‐based luminescence probe (Ru‐NBD) for discrimination and detection of biothiols in vitro and in vivo (Scheme 1A). Ru‐NBD is designed with a “luminophore‐responsive linker‐luminophore” approach, that is, linking two different luminophore, i.e., Ru(II) complex and NBD fluorophore through a responsive “O” ether bond linker (Scheme 1B). Due to the strong electron withdrawing effect of NBD, the Ru(II) complex phosphorescence is expected to be quenched through a PET process from Ru(II) complex to NBD. For the fluorescence of NBD moiety, it has been reported that the emission of this ICT‐based fluorophore is dominated by electron‐donating ability of substitution groups. The NBD‐OR and NBD‐SR are nonfluoresent while the NBD‐NR with strong electron‐donating substitution group (‐NR) shows intense fluorescence (relative quantum yields in methanol NBD‐NR, 62.7%; NBD‐SR, 0.6%, and NBD‐OR, 0.0%).^[^ ^18^ ^]^ Therefore, it is reasonable to speculate that the Ru‐NBD is weakly luminescent in the absence of biothiols.

As shown in Scheme 1B, in the presence of biothiols, the ether bond of Ru‐NBD is expected to be rapidly cleaved through a nucleophilic substitution reaction (S_N_AR),^[^ ^18^, ^19^ ^]^ affording red emitting Ru‐OH and nonfluorescent NBD‐SR. For the products of the reaction Ru‐NBD with Cys/Hcy, NBD‐SR2 can further undergo a five‐ or six‐member cyclic intermediate‐associated rearrangement to form corresponding highly fluorescent NBD‐NR. While similar rearrangement of NBD‐SR1, the product of the reaction between Ru‐NBD and GSH is unavailable. As a result, discrimination of GSH and Cys/Hcy is feasible through monitoring steady‐state luminescence at both red and green channels, *i.e*, turn on both red and green emission of Ru‐NBD upon addition of Cys/Hcy while turn on red emission only in the presence of GSH (Scheme 1A,B). Through introducing a delay time, the TGL analysis allows elimination of background autofluorescence and short lifetime NBD green fluorescence, ensuring the detection of total biothiols via recording long lifetime Ru(II) complex red signal (Scheme 1A).

Density function theory (DFT) and time‐dependent DFT (TD‐DFT) calculations were first conducted to understand the electronic transitions and rationalize the PET‐mediated emission characters of Ru‐NBD and Ru‐OH. The molecular geometries of both complexes at ground state (S_0_) and lowest‐lying excited state (T_1_) were optimized (Figures S1 and S2, Tables S1 and S2, Supporting Information). In all calculations, polarizable continuum model (PCM) was employed in considering the effects of water (H_2_O) as the solvent. With the optimized S_0_ molecular geometries of Ru‐NBD and Ru‐OH, the corresponding highest‐occupied molecular orbitals (HOMOs) and the lowest‐unoccupied molecular orbitals (LUMOs) were then calculated. As shown in Figure S3 in the Supporting Information, Ru‐NBD exhibits Ru(II) center characters for HOMO, HOMO‐1, and HOMO‐2. HOMO‐3 of Ru‐NBD is mainly located on the NBD‐bpy with small distribution on the Ru(II) center. LUMO and LUMO+1 of Ru‐NBD are fully distributed on NBD moiety rather than whole bpy ligand, while LUMO+2, LUMO+3, and LUMO+4 of Ru‐NBD are mainly located on the other two bpy ligands. In Ru‐OH, HOMOs are dominated by Ru(II) center with a small distribution on OH‐bpy ligand, and LUMOs are largely distributed on three bpy ligands (Figure S4, Supporting Information).

The absorption profiles of Ru‐NBD and Ru‐OH were then examined through the investigation of electronic transitions on the basis of TD‐DFT calculations (Table S3, Supporting Information). In Ru‐NBD, electronic transitions S_0_ to the first excited state (S_1_) is dominated by HOMO → LUMO (99.13%) with typical electron transfer (ET) character. In addition, Ru‐NBD's states of S_2_, S_3_, S_7_ and S_10_ also exhibit ET character from Ru(II) center to NBD moiety. In contrast, Ru‐OH's excitation states of S1‐S10 are dominated with characteristic ^1^MLCT transitions. The calculated excitation energy (2.61 eV) and the corresponding wavelength (474.74 nm) are in agreement with characteristic absorption in the visible range of Ru(II) complex.

TD‐DFT calculations were then conducted to rationalize the luminescence “OFF‐ON” response of Ru(II) complexes. Based on the optimized molecular geometries of Ru‐NBD and Ru‐OH at the lowest‐lying triplet excited state (T_1_), MOs of both complexes were calculated. As shown in Figures S5 and S6 and Table S4 in the Supporting Information, HOMOs and LUMOs of Ru‐NBD and Ru‐OH at T_1_ exhibit similar distribution with the one calculated on the basis of optimized S_0_ molecular geometries. The T_1_ of Ru‐NBD is dominated by HOMO → LUMO (97.36%), and T_1_‐T_3_ display clear ET character from Ru(II) center to NBD moiety. In Ru‐OH, T_1_ is also mainly contributed by HOMO → LUMO (86.35%), and T_1_‐T_3_ exhibit clear ^3^MLCT character that is generally featured by luminescent Ru(II) complexes. The calculated emission energy (1.87 eV) and the corresponding wavelength (661.69 nm) are in agreement with characteristic emission of Ru(II) complex (Ru‐OH, *λ*_em_ = 628 nm). As the result of these computations, it is clearly that the luminescence of Ru‐NBD probe can be switched on after reacting with biothiols to yield Ru‐OH.

To futher investigate the driving force of the ET process from Ru(II) center to NBD, the thermodynamic value (free energy change of the PET effect), ∆*G_PET_*, was determined by both theroretic computation and electrochemical approaches using the Rehm‐Weller equation. With theoretical computation of Ru‐NBD, the Δ*G_PET_* is determined to be −91.18 kJ mol^−1^ (‐−0.945 eV). From the cyclic voltammograms (CVs) of Ru‐OH (Figure S7A, Supporting Information) and NBD‐Cl (Figure S7B, Supporting Information), the oxidation of Ru‐OH occurs at a potential of 1.106 V (vs Ag/AgNO_3_), and the one‐electron reduction of the NBD‐Cl moiety occurs around −0.761 V (vs Ag/AgNO_3_). The change of ∆*G*_PET_ is determined to be be −37.92 kJ mol^−1^ (−0.393 eV). The negative value of Δ*G_PET_* indicates the thermodynamical possibility of ET from Ru(II) center to NBD moiety.

Following the theoretical computation, the Ru‐NBD was readily synthesized by a two‐step procedure (Scheme S1, Supporting Information). The chemical structure of Ru‐NBD was confirmed by ^1^H NMR, ^13^C NMR, high‐resolution mass spectrometry (ESI‐HRMS), and elemental analysis (Figures S8–S10, Supporting Information). The products of the reactions of Ru‐NBD with GSH, Cys, and Hcy were then studied by ESI‐MS (positive model) analysis. As shown in Figure S11 in the Supporting Information, the molecular ionic peaks of Ru‐NBD were absent and new peaks at *m*/*z* = 807.17 ([M‐PF_6_]^+^), 330.89 ([M‐2PF_6_]^2+^) were observed, which are assigned to the molecular ionic peaks of Ru‐OH. ESI‐MS (negative model) analysis of the reaction products of Ru‐NBD with GSH, Cys, and Hcy showed molecular ionic peak at *m*/*z* = 283.1 ([NBD‐NR1 – H]^−^) (Figure S12, Supporting Information), 297.0 ([NBD‐NR2 – H]^−^) (Figure S13, Supporting Information) and 469.1 ([NBD‐SR – H]^−^) (Figure S14, Supporting Information), respectively, indicating the formation of NBD‐NR and NBD‐SR products.

### 2.2 Steady‐State Luminescence Discrimination and Detection of Biothiols

**Figure 1:**
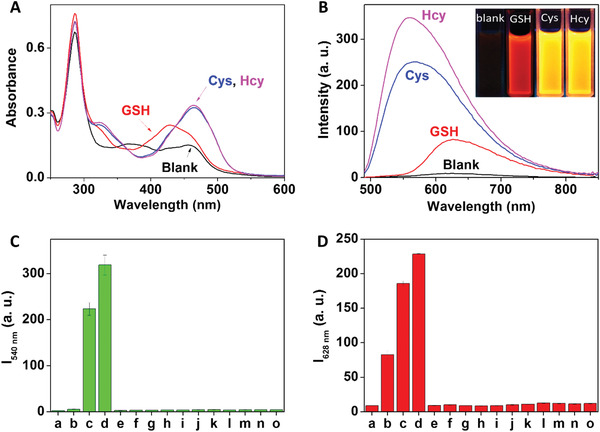
Spectrometric response of Ru‐NBD to biothiols. A) Absorption and B) steady‐state emission changes of Ru‐NBD (10 × 10^−6^ m) in the absence and the presence of GSH, Cys, and Hcy in 50 × 10^−3^ m Tris‐HCl buffer of pH 7.4 (B inset: photo of luminescence color changes of Ru‐NBD in the absence and presence of GSH, Cys and Hcy). Emission intensity of Ru‐NBD (10 × 10^−6^ m) at C) 540 nm and D) 628 nm after reacted with different amino acids (200 × 10^−6^ m) in 50 × 10^−3^ m Tris‐HCl buffer of pH 7.4. Amino acids: a) blank, b) GSH, c) Cys, d) Hcy, e) tryptophan, f) threonine, g) glycine, h) valine, i) leucine, j) histidine, k) proline, l) serine, m) tyrosine, n) alanine, and o) aspartic acid.

The UV‐vis absorption spectra of Ru‐NBD and its reaction products with different biothiols were measured in 50 × 10^−3^ m Tris‐HCl buffer of pH 7.4. As shown in **Figure** **1**A, all solutions displayed identical absorption maximum at ≈290 nm, which can be assigned to the intraligand *π*→π* transition of the bpy ligands (intraligand charge transfer, ILCT). A broad absorption band centered at about 460 nm was also observed for Ru‐NBD because of the ^1^MLCT of Ru(II) complex. Interestingly, absorbance of this visible absorption band was increased and the peak was shifted to about 480 nm in the presence of Cys/Hcy, which is clearly different with the one of Ru‐NBD reacting with GSH. The increase and the bathochromic shift of UV‐vis spectra are attributed to the formation of NBD‐NR after the reaction between Ru‐NBD and Cys/Hcy, where the new NBD‐NR absorption band centered at about 480 nm emerged and overlapped with the MLCT absorption of Ru‐OH.

The luminescence properties of Ru‐NBD were then evaluated in the absence and presence of biothiols in 50 × 10^−3^ m Tris‐HCl buffer of pH 7.4. As expected, Ru‐NBD showed weak luminescence with emission peak at 628 nm due to the effective PET process (*ϕ* = 0.056%) (Figure 1B). This emission was increased after Ru‐NBD reacting with GSH due to the formation of red‐emitting Ru‐OH (*ϕ* = 2.31%). In sharp contrast, the red‐emitting luminescence was increased and a new emission peak at 540 nm was observed in the presence of either Cys or Hcy. Under UV lamp, clearly yellow luminescence color of the solution was observed which can be attributed to the overlap between green emission of NBD‐NR and red emission of Ru‐OH (Figure 1B inset).

The capability of Ru‐NBD for specific response to biothiols over other amino acids was then examined. Ru‐NBD in Tris‐HCl buffer (50 × 10^−3^ m, pH 7.4) was treated with various amino acids, including tryptophan, threonine, glycine, valine, leucine, histidine, proline, serine, tyrosine, alanine, and aspartic acid. As shown in Figure S15 in the Supporting Information, the emission spectra and luminescence color of Ru‐NBD were not changed in the presence of various amino acids other than GSH, Cys, and Hcy. Luminescence intensity at both 540 (Figure 1C) and 628 nm (Figure 1D) of Ru‐NBD significantly increased upon the addition of Cys and Hcy, while emission enhancement was observed at 628 nm in the presence of GSH. Together with the different luminescence response mechanism of Ru‐NBD to GSH and Cys/Hcy, the results of selectivity experiments indicate that Ru‐NBD is capable of discriminating GSH and Cys/Hcy from other amino acids.

Conditions for luminescent biothiols detection were then optimized by considering the reaction time and the effect of pH on the luminescence response. Figure S16 in the Supporting Information shows the time‐profile luminescence response of Ru‐NBD to different biothiols in 50 × 10^−3^ m Tris‐HCl buffer of pH 7.4. Ru‐NBD shows weak and stable luminescence emission, while rapid luminescence response was obtained after incubation of Ru‐NBD with GSH, Cys and Hcy, respectively. Maximum luminescence intensity was obtained within 15 min incubation, indicating that the nucleophilic substitution reaction (Ru‐NBD reacting with biothiols to yield Ru‐OH and NBD‐SR) and rearrangement of NBD‐SR to yield NBD‐NR can be completed within 15 min. Figure S17 in the Supporting Information illustrates the luminescence intensity changes of Ru‐NBD in the absence and presence of biothiols at 540 and 628 nm in Tris‐HCl buffer with different pH values. The emission of Ru‐NBD at these two wavelengths is pH independent. Remarkable luminescence response at both 540 and 628 nm was noticed in the pH around 7–8. Considering the fact that physiologically intracellular pH is most commonly between 7.0 and 7.4 (except lysosomes with pH from around 4.5 to 6), the results of pH effects on the luminescence response indicate that Ru‐NBD could be used as a probe for biothiols detection under the physiological pH condition.

**Figure 2:**
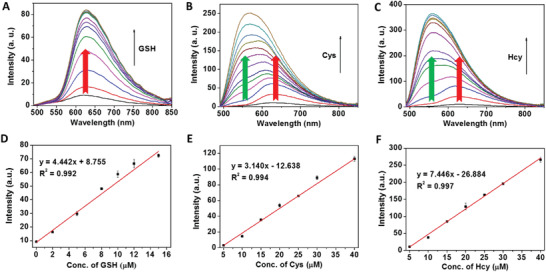
Emission spectra of Ru‐NBD (10 × 10^−6^ m) in the absence and presence of different concentrations of GSH A) (0 × 10^−6^, 2 × 10^−6^, 5 × 10^−6^, 8 × 10^−6^, 10 × 10^−6^, 12 × 10^−6^, 15 × 10^−6^, 20 × 10^−6^, 30 × 10^−6^, 50 × 10^−6^, 100 × 10^−6^, 150 × 10^−6^, and 200 × 10^−6^ m), B) Cys (0 × 10^−6^, 5 × 10^−6^, 10 × 10^−6^, 15 × 10^−6^, 20 × 10^−6^, 25 × 10^−6^, 30 × 10^−6^, 40 × 10^−6^, 60 × 10^−6^, 80 × 10^−6^, 100 × 10^−6^, 150 × 10^−6^, and 200 × 10^−6^ m) and Hcy (0 × 10^−6^, 5 × 10^−6^, 10 × 10^−6^, 15 × 10^−6^, 20 × 10^−6^, 25 × 10^−6^, 30 × 10^−6^, 40 × 10^−6^, 60 × 10^−6^, 80 × 10^−6^, 100 × 10^−6^, 150 × 10^−6^, and C) 200 × 10^−6^ m in 50 × 10^−3^ m Tris‐HCl buffer (pH 7.4). Linear correlation of emission intensity at D) 628 nm and 540 nm E,F) against the concentration of D) GSH, E) Cys, and F) Hcy.

Capability of Ru‐NBD in quantitative detection of GSH and Cys/Hcy was then evaluated by steady‐state luminescence analysis. As shown in **Figure** **2**A, an enhancement of luminescence spectra was noticed upon increasing GSH concentrations. A good linearity relationship at 628 nm was obtained by plotting the luminescence intensity against GSH concentrations (Figure 2D). The detection limit for GSH was calculated to be 138.9 × 10^−9^ m based on the concentration corresponding to three standard deviations of the background signal (DOL = 3*σ*/k). Upon addition of increasing amount of Cys/Hcy, emission spectra were clearly enhanced along with the emergence of new emission band centered at 540 nm (emission of NBD‐NR) (Figure 2B,C). Based on the luminescence intensity at 540 nm (Figure 2E,F), the detection limits for Cys and Hcy were determined to be 196.4 and 82.9 × 10^−9^ m, respectively. The results of luminescence analysis suggest that Ru‐NBD can serve as a luminescence probe for discrimination and quantitative detection of GSH and Cys/Hcy.

### 2.3 Time‐Gated Luminescence Detection of Total Biothiols

**Figure 3:**
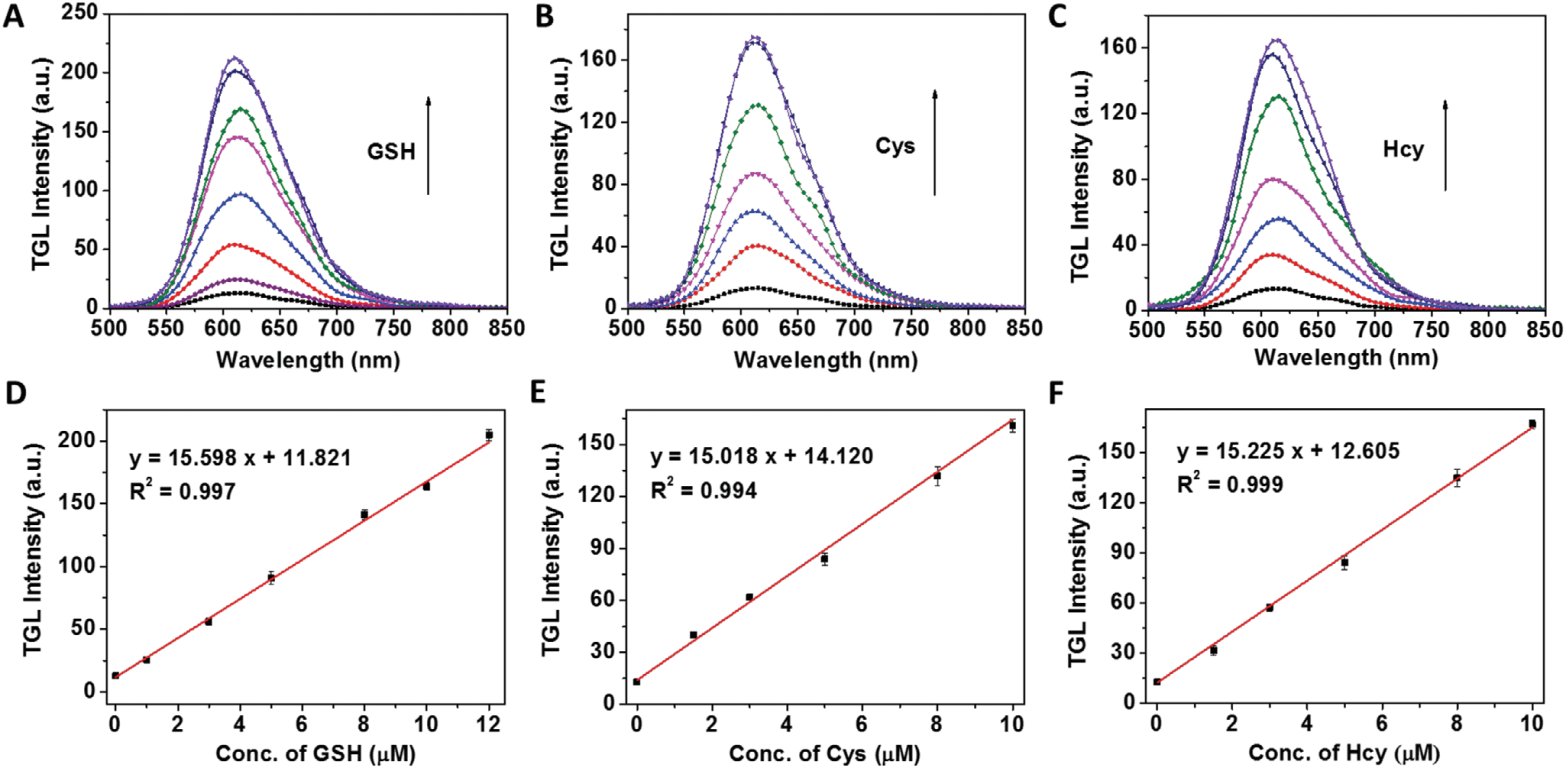
Time‐gated emission spectra (delay time: 100 ns) of Ru‐NBD (10 × 10^−6^ m) in the absence and presence of different concentrations of GSH (0–15 × 10^−6^ m) A), Cys (0–15 × 10^−6^ m) B) and Hcy (0–15 × 10^−6^ m) C) in 50 × 10^−3^ m Tris‐HCl buffer (pH 7.4). Linear correlation of emission intensity at 610 nm against the concentration of D) GSH, E) Cys, and F) Hcy. (*λ*_ex_ = 450 nm)

TGL is a promising analysis technique that can efficiently eliminate autofluorescence noise to provide background‐free luminescence signal for biothiols detection. As shown in Figure S18 in the Supporting Information, luminescence emission lifetime of Ru‐NBD, Ru‐OH and NBD‐NR1 in Tris‐HCl buffer (50 × 10^−3^ m, pH 7.4) was determined to be 318.68, 301.95, and 0.80 ns, respectively. The long lifetime of Ru(II) complex probe enables background‐free TGL detection of total biothiols upon introducing a delay time post excitation. **Figure** **3** displays the TGL emission spectra of Ru‐NBD in the presence of increasing contraction of GSH, Cys, and Hcy, respectively. TGL was clearly enhanced and the emission intensity showed biothiols concentration dependent. In comparison with steady‐state luminescence spectra (Figure 2), short lifetime emission of NBD‐NR was effectively eliminated in the TGL mode. As shown in Figure 3D–F, TGL intensities exhibit good linearity against the concentration of GSH, Cys and Hcy, respectively. The detection limits for GSH, Cys and Hcy were determined to be 83.32 × 10^−9^, 86.54 × 10^−9^, and 85.36 × 10^−9^ m, respectively. More importantly, the slope and intercept of these three linear equations are similar, suggesting Ru‐NBD is able to detect total biothiols using TGL analysis.

Proof‐of‐concept experiments were then performed to validate the capability of “two birds with one stone” probe (Ru‐NBD) for discrimination and detection of biothiols. For a mixture containing GSH (8 × 10^−6^ m) and Cys (8 × 10^−6^ m), the concentration of total biothiols (*C_total_*) was determined to be 16.56 ± 0.53 × 10^−6^ m by TGL analysis, and Cys concentration (*C_Cys_*) was determined to be 9.05 ± 0.23 × 10^−6^ m by steady‐state luminescence analysis. Therefore, the concentration of GSH (*C_GSH_*) was thus calculated (*C_total –_ C_Cys_*) to be 7.51 ± 0.31 × 10^−6^ m. Upon further addition of GSH and Cys to the mixture, the concentrations of Cys, GSH and total biothiols were detected and the recovery was determined to be in the range of 85.86–113.12% (Figure S19 and Table S5, Supporting Information), indicating the high accuracy of Ru‐NBD for discrimination and detection of biothiols. In contrast, quantitative detection of total biothiols and discrimination of GSH and Cys/Hcy are impossible without TGL analysis because red channel signal for total biothiols determination was produced by both Ru‐OH and the overlapped emission of NBD‐NR.

### 2.4 Visualization of Biothiols in Live Cells

Prior to establish the biological application of Ru‐NBD, cytotoxicity of Ru‐NBD was assessed by MTT assay. As shown in Figure S20 in the Supporting Information, the cell viability was greater than 88% after incubation of 200 × 10^−6^ m Ru‐NBD for 24 h, indicating that Ru‐NBD is limited cytotoxicity to live cells.

**Figure 4:**
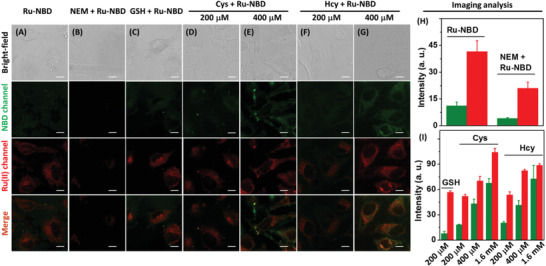
Luminescence imaging of biothiols in HeLa cells. A) HeLa cells were incubated with Ru‐NBD (50 × 10^−6^ m) for 5 h. B) HeLa cells were incubated with NEM (100 × 10^−6^ m) for 2 h before staining with Ru‐NBD (50 × 10^−6^ m) for another 5 h. C–G) HeLa cells were incubated with GSH (200 × 10^−6^ m), Cys (200 × 10^−6^ m), Cys (400 × 10^−6^ m), Hcy (200 × 10^−6^ m), Cys (400 × 10^−6^ m), respectively, followed by the staining with Ru‐NBD (50 × 10^−6^ m) for another 5 h. Intracellular luminescence intensity at both green and red channels of H) group A and B cells and I) group C‐G and HeLa cells supplied with 1600 × 10^−6^ m Cys and Hcy, respectively. Scale bar: 10 µm.

To evaluate the feasibility of Ru‐NBD for imaging of biothiols in live cells, HeLa cells were treated with 50 × 10^−6^ m Ru‐NBD for 5 h, followed by the luminescence imaging at both red Ru(II) and green NBD channels. As shown in **Figure** **4**A, red and weak green emission in HeLa cells were observed after incubation with Ru‐NBD, while HeLa cells in the control group did not show any emission at both red and green channels (Figure S21, Supporting Information). Pretreatment of the HeLa cells with NEM (N‐Ethylmaleimide) remarkably suppressed the intracellular luminescence (Figure 4B,H) because part of biohtiols was removed by reacting with NEM. Supplying HeLa cells with GSH increased the intracellular luminescence at red Ru(II) channel, while green channel emission was negligibly changed (Figure 4C,I). Interestingly, pretreatment of HeLa cells with Cys and Hcy elevated the intensity intracellular luminescence at both red and green channels, and the intensity of both channel showed clearly Cys/Hcy‐concentration dependent (Figure 4D–G,I; Figure S22, Supporting Information). As the fact of high level biothiols in HeLa cells, these images indicate the feasibility of Ru‐NBD for imaging endogenous biothiols level variations and its potential in discrimination of GSH and Cys/Hcy in live cells.

The feasibility of Ru‐NBD for TGL imaging was then investigated through visualisation of intracellular biothiols in live HeLa cells. The cells were firstly supplied with 400 × 10^−6^ m Cys/Hcy and then incubated with Ru‐NBD for TGL imaging. As shown in Figure S23 in the Supporting Information, obvious luminescence at both green and red channel was observed for the HeLa cells stained with Ru‐NBD (Figure S23A, Supporting Information). Introducing of a delay time (4 ns) to NBD channel remarkably decreased intracellular green luminescence intensity to 4.47% (Cys treated group) and 5.51% (Hcy treated group), while the signal of Ru(II) channel remained constant value without time‐gating (Figure S23G,H, Supporting Information). With images time delayed for 4 ns, over 60% signal of Ru(II) complex red channel luminescence retained (Figure S24, Supporting Information), which is in sharp contrast with the TGL imaging of green NBD channel (Figure S23G,H, Supporting Information).

**Figure 5:**
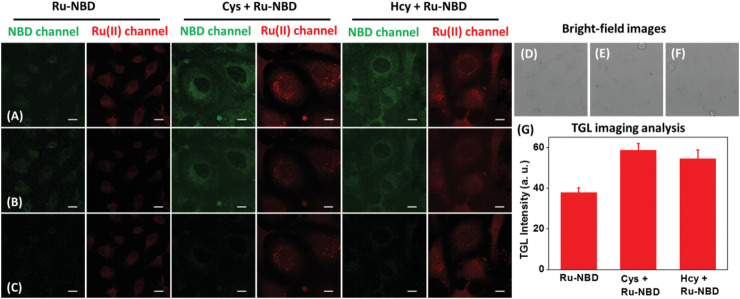
Time‐gated luminescence imaging of biothiols in live HeLa cells. HeLa cells (Cys/Hcy pretreated HeLa cells) were incubated with Ru‐NBD (50 × 10^−6^ m) for 5 h, followed by the time‐gated confocal microscopy imaging. The signals from green and red channel were recorded within A) 0–12 ns ; B) 0–4 ns ; and C) 4–12 ns. Bright field imaging of D) Ru‐NBD, E) Cys + Ru‐NBD, and F) Hcy + Ru‐NBD. G) Change of intracellular red channel signal of each group. Scale bar: 20 µm.

As the facts of the retained red channel Ru(II) complex signal and removed green channel NBD signal, TGL imaging of intracellular total biothiols was readily to be demonstrated. As shown in **Figure** **5**, with the imaging time delayed 4 ns, the obtained red emission signal is attributed to the Ru‐OH (reaction product between Ru‐NBD and intracellular biothiols). Supplying HeLa cells with exogenous Cys/Hcy (400 × 10^−6^ m) increased the TGL signal of Ru(II) channel (Figure 5G), indicating the potential of Ru‐NBD for TLG imaging of total biothiols changes in live cells.

After the reaction of biothiols with internalized Ru‐NBD in live HeLa cells, the productions of Ru‐OH, NBD‐SR1, and NBD‐NR were confirmed by ESI‐MS analysis of cell extraction solution. The HeLa cells were incubated with Ru‐NBD for 5 h at at 37 °C in a 5% CO_2_/95% air incubator. After washing with PBS for three times, the cells were collected and lysed by sonication. The cell extraction solution was then collected for ESI‐MS analysis. As shown in Figure S25 in the Supporting Information, MS peaks at *m*/*z* = 283.2, 296.9, 468.9, and 330.7 were observed, which are assigned to the molecular ionic peaks of [NBD‐NR1‐H]^−^, [NBD‐NR2‐H]^−^, [NBD‐SR1‐H]^−^, and [Ru‐OH‐2PF6]^2+^, respectively. The results of MS analysis indicate the production of NBD‐NR1, NBD‐NR2, NBD‐SR1, and Ru‐OH in HeLa cells after the reaction of Ru‐NBD with Cys, Hcy, and GSH.

### 2.5 Imaging of Biothiols In Vivo

**Figure 6:**
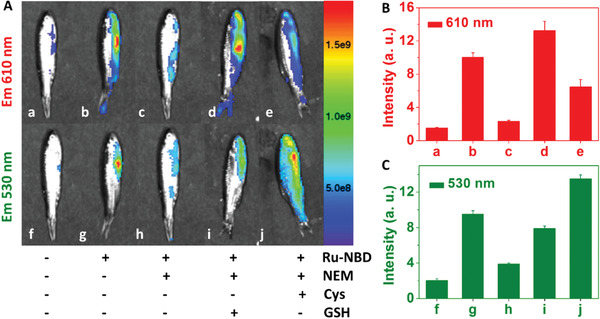
Luminescence imaging of biothiols in adult zebrafish using Ru‐NBD as the probe. A: a,f) Control group; b,g) zebrafish stained with Ru‐NBD (200 × 10^−6^ m); c, h) zebrafish was treated with NEM (500 × 10^−6^ m), then incubated with Ru‐NBD (200 × 10^−6^ m); zebrafish was treated with NEM (500 × 10^−6^ m), followed by supplying with GSH (500 × 10^−6^ m) d,i) or Cys (500 × 10^−6^ m) e, j) before staining with Ru‐NBD (200 × 10^−6^ m). Mean luminescence intensity of zebrafish at different conditions, red channel (B, *E*_m_ = 610 nm filter); green channel (C, *E*_m_ = 530 nm filter).

Visualization of biothiols in adult zebrafish was then performed using Ru‐NBD as the luminescence probe. Under excitation of 480 nm LED light, adult zebrafish showed almost no luminescence at both red (*E*_m_ 610 nm filter) and green (*E*_m_ 530 nm filter) channels (**Figure** **6**a,f), while displayed clear signal from both channels after incubating with Ru‐NBD (Figure 6b,g). The emergence of the luminescence from both channels is attributed to the response of Ru‐NBD to endogenous biothiols in zebrafish. This result was further confirmed by image that weak luminescence could be observed after scavenging of biothiols by NEM treatment (Figure 6c,h). Furthermore, higher luminescence intensities can be observed at red and green channel when zebrafish were supplied with GSH and Cys, respectively (Figure 6B,C). To confirm the biocompatiblity of Ru‐NBD in vivo, respiratory rate of zebrafish was recorded before and after incubation with 200 × 10^−6^ m Ru‐NBD. The respiratory rate of zebrafish was around 150 beats per minute after incubation with Ru‐NBD for 2 days. The rate is similar to the control group and literature reports,^[^ ^20^ ^]^ demonstrating good biocompatibility of Ru‐NBD in vivo. These results reveal that Ru‐NBD is suitable for visualization and discrimination biothiols in zebrafish.

**Figure 7:**
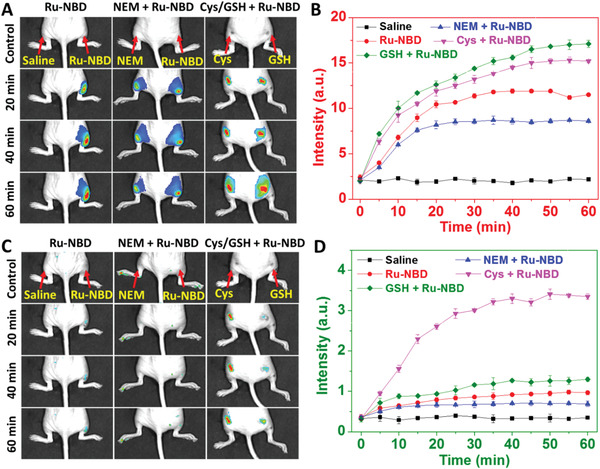
A,C) Luminescence imaging of biothiols in live mice using Ru‐NBD as a probe. Group 1: 50 µL saline and Ru‐NBD (500 × 10^−6^ m) were subcutaneously injected into the left and right hind limbs, respectively. Group 2: 50 µL NEM and normal saline were subcutaneously injected into the left and right hind limbs, respectively, followed by the injection of 50 µL Ru‐NBD (500 × 10^−6^ m) into the both hind limbs. Group 3: 50 µL GSH (500 × 10^−6^ m) and Cys (500 × 10^−6^ m) were injected into the left and right legs, respectively, followed by the injection of 50 µL Ru‐NBD (500 × 10^−6^ m) into the both hind limbs. Time‐dependent enhancement of mean luminescence intensities of images, red channel (B, *E*_m_ = 610 nm filter); green channel (D, *E*_m_ = 530 nm filter).

Time‐dependent luminescence imaging of biothiols in live mice was then validated using Ru‐NBD as a probe. As shown in **Figure** **7**, obvious luminescence increases from red and green channels were noticed within 30 min upon subcutaneous injection of Ru‐NBD (Figures S26 and S27, Supporting Information), while the intensities were decreased for the group that pretreated with NEM (Figures S28 and S29, Supporting Information). Injection of exogenous Cys into left hind limbs, significant luminescence increases at both channels were also noticed within 30 min post Ru‐NBD injection (Figure 7B,D). Different with the luminescence changes of Cys supplying group, presupplying of GSH led to further increase of the luminescence signal of red channel (Figure S30, Supporting Information). In comparison, slight increase of luminescence intensity of the green channel were observed in the same region (Figure S31, Supporting Information). The images indicate that Ru‐NBD can be used as an effective luminescence probe to monitor the changes of biothiols concentrations in mice.

## 3 Conclusion

In present work, a novel “two birds with one stone” ruthenium(II) complex probe, Ru‐NBD, has been developed for biothiols detection and discrimination in vitro and in vivo. Ru‐NBD (“stone”) is successfully used for the detection of total biothiols (“bird” one) by TGL analysis, and discrimination of GSH and Cys/Hcy (“bird” two) through steady‐state luminescence analysis. Results of steady‐state and TGL analyses demonstrated the applicability of Ru‐NBD for the highly sensitive, selective quantification and discrimination of biothiols in vitro. Furthermore, Ru‐NBD shows good biocompatibility and good cell membrane penetrability, allowing it to be used as the probe for luminescence imaging and discrimination of biothiols in biological organisms. With this “two birds with one stone” probe, visulisation total biothiols and discrimination of GSH and Cys/Hcy in live cells through TGL and steady‐state luminescence analysis were successfully demonstrated. In imaging of biothiols in live adult zebrafish and mice, different changes of luminescence signal of Ru‐NBD were obtained in the presence of exogenous GSH and Cys, indicating the capability of Ru‐NBD in visualization and discrimination of biothiols in vivo. The success of this “two birds with one stone” probe not only provided a useful approach for future biomedical researches of biothiols in live organisms but also formed solid knowledge base for the future studies on the transitional metal complexes in biomedical applications.

## Conflict of Interest

The authors declare no conflict of interest.

## Supporting information

Supporting Information
