# Supplementary material for: “Two Birds with One Stone” Ruthenium(II) Complex Probe for Biothiols Discrimination and Detection In Vitro and In Vivo
Source: Adv Sci (Weinh). 2020 May 29;7(14):2000458. doi: 10.1002/advs.202000458 (PMC7375222; doi:10.1002/advs.202000458)
Supplement: Supplementary file 1 — Supporting Information [file ADVS-7-2000458-s001.pdf]

Copyright WILEY-VCH Verlag GmbH & Co. KGaA, 69469 Weinheim, Germany, 2020.

Supporting Information

"Two Birds with One Stone" Ruthenium(II) Complex Probe for Biothiols Discrimination and Detection in Vitro and in Vivo

Chaolong Liu, Jianping Liu, Wenzhu Zhang, Yong-Lei Wang, Qi Liu, Bo Song, Jingli Yuan, Run Zhang

C. Liu, Dr. W. Zhang, Q. Liu, Dr. B. Song, Prof. J. Yuan
State Key Laboratory of Fine Chemicals, School of Chemical Engineering, Dalian University of Technology, Dalian 116024, China.
E-mail: wzhzhang@dlut.edu.cn, jlyuan@dlut.edu.cn

J. Liu, Dr. R. Zhang
Australian Institute for Bioengineering and Nanotechnology, The University of Queensland, St. Lucia, QLD 4072, Australia.
E-mail: r.zhang@uq.edu.au

Dr. Y.-L. Wang
Department of Materials and Environmental Chemistry, Stockholm University, Stockholm, SE-10691, Sweden

Experimental section

Reagents and instruments.

Ru-OH was synthesized by following the procedure reported previously.[1] NBD-Cl and BBr_3 were purchased from Aladdin. GSH, Cys, Hcy, Roswell Park Memorial Institute's Medium (RPMI-1640), fetal bovine serum (FBS), *L*-glutamine, penicillin and 3-(4,5-dimethylthiazol-2-yl)-2,5-diphenyltetrazolium bromide (MTT) were purchased from Life Technologies. Unless otherwise stated, all chemical materials were purchased from commercial sources and used without further purification. Deionized distilled water was used throughout.

^{1}H NMR and ^{13}C NMR spectra were recorded on a Bruker Avance spectrometer (500 MHz for ^{1}H and 100 MHz for ^{13}C). Mass spectra were obtained using a HP1100 LC/MSD mass spectrometer. Elemental analysis was carried out on a Vario-EL analyser. Absorption spectra were measured on a Perkin-Elmer Lambda 35 UV-vis spectrometer. The measurements on the luminescence features were performed using an Edinburgh FS 50 luminescence spectrometer with excitation and emission slits of 2 nm. Confocal luminescence and time-gated luminescence imaging experiments of biothiols in live HeLa cells were carried out on a Leica SP8 laser-scanning microscope. The image analysis was performed by ImageJ software version 1.44p. Imaging of biothiols in adult zebrafish and mouse was performed on a SPECTRAL Ami Imaging Systems (Spectral Instruments Imaging, LLC, Tucson, AZ) with an excitation filter of 480 nm and an emission filter of 610 nm and 530 nm. Nude mice (6-8 weeks) and zebrafish were obtained from the Experimental Animal Center of the Dalian Medical University, China, and used according to guidelines of the Institutional Animal Care that was approved by the Animal Ethical and Welfare Committee (AEWC) of Dalian Medical University. All the data were calculated using the region of interest (ROI) function of Amiview Analysis software (Version 1.7.06), and values are presented as the mean ± SD for each group of three experiments.

Theoretical calculation

The theoretical computation was conducted by using the Gaussian 09 package of programs.[2] The molecular structures of **Ru-NBD** and **Ru-OH** at ground-states and excited states were firstly optimized using the density functional theory (DFT). Based on these optimized molecular structures, the related excited-state calculations were then conducted by the time-dependent DFT (TD-DFT) method. The Beck's three-parameter hybrid functional with the Lee-Yang-Parr correlation functional (B3LYP) was used throughout. The LanL2DZ basis set[3] was used for Ru atoms in both complexes, whereas 6-311*G(d, p) basis set[4] was applied to hydrogen, carbon, nitrogen, sulphur and oxygen atoms. The polarized continuum model (PCM)[5] was employed for the solvent effects (water) in optimization, absorbance and emission calculations.

Evaluate the PET effect by thermodynamics calculation

The thermodynamics value ΔG_{PET}, can be determined by calculation using the Rehm-Weller equation,[6]

$$\Delta G_{PET} = E^{ox}\,(D^{+}/D) - E^{red}(A/A^{-}) - \Delta E_{0,0} - w_p$$

where $E^{ox}\,(D^{+}/D)$ and $E^{red}(A/A^{-})$ are the oxidation and reduction potentials of the electron donor and acceptor, respectively. Herein, these values are approximated as the energy difference of HOMO and LUMO, which can be obtained from DFT calculations. $\Delta E_{0,0}$ is the singlet excited energy, which can be approximately calculated as the crossing point of excitation and emission spectra of **Ru-NBD**. w_p is the work term for the charge separation state, which can be obtained by equation,

$$w_p = e^2/4\pi\varepsilon R$$

where ε is the dielectric constant of media, i.e. water in this work with ε value of 80.36 at 20ºC; R is the distance between electron donor and acceptor, *i. e.* distance between NBD and bpy, which is approximated to the distance between NO_2 group of NBD moiety and N atoms of bpy

ligand (calculated by the ground state geometry of **Ru-NBD**).

Synthesis and characterization of Ru-NBD

The synthesis procedure of compound **Ru-NBD** is illustrated in Scheme S1 and corresponding synthesis procedures were described below:

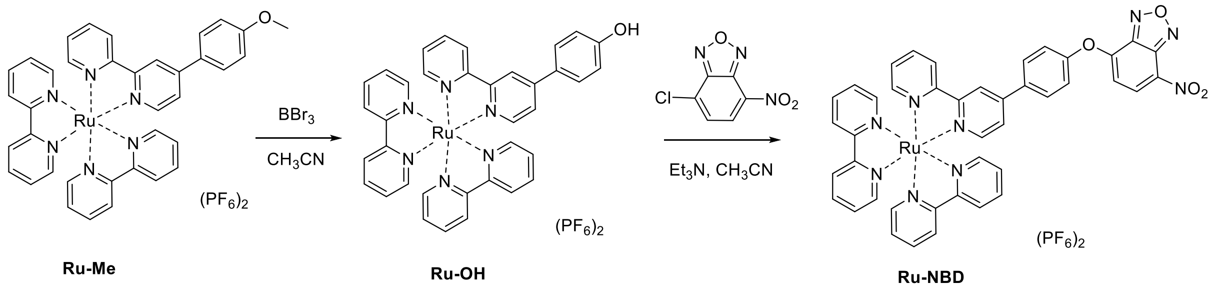

Scheme S1. Synthesis procedure of **Ru-OH** and **Ru-NBD.**

To a solution of **Ru-OH** (67 mg, 0.07 mmol) in dry CH_3CN (20 mL), NBD-Cl (70 mg, 0.35 mmol), Et_3N (15 mg, 0.15 mmol) was added, and the mixture was stirred at room temperature for 5 h. The solvent was removed by rotary evaporation, and then the crude product purified by silica gel column chromatography using CH_3CN-H_2O-KNO_3 (sat.) (100:5:1, v/v/v) as eluent. The product was dissolved in 10 mL anhydrous CH_3CN and filtered to remove excess KNO_3. After removal of the solvent, the product was dissolved in a small amount of deionized water. Then, a saturated aqueous solution of NH_4PF_6 was added to give a red precipitate. The product was filtered, and washed with a small amount of cold water. **Ru-NBD** was obtained as a red solid (60 mg, 77% yield). ^1H NMR (500 MHz, CD_3CN): δ = 8.76 (s, 1H), 8.68 (d, *J* = 10.5 Hz, 1H), 8.47-8.57 (m, 5H), 8.00-8.14 (m, 7H), 7.81 (d, *J* = 6 Hz, 1H), 7.72-7.79 (m, 5H), 7.67 (d, *J* = 6.5 Hz, 1H), 7.53 (d, *J* = 10.5Hz, 2H), 7.37-7.47 (m, 5H), 6.78 (d, *J* = 10.5 Hz, 1H). ^{13}C NMR (100 MHz, CD_3CN): 157.22, 156.72, 156.66, 154.89, 152.67, 151.42, 151.35, 147.83, 145.39, 144.41, 137.54, 137.46, 134.08, 133.89, 131.12, 129.59, 127.31, 127.27, 124.51, 123.98, 121.54, 121.35, and 109.88. ESI-HRMS (m/z): calcd. for $C_{42}H_{29}F_6N_9O_4PRu$: 970.1028, found: 970.0998 ($[M-PF_6]^+$); calcd. for $C_{42}H_{29}N_9O_4Ru$: 412.5693, found: 412.5679 ([M-

$2PF_6]^{2+}$). Elemental analysis calcd. (%) for $C_{42}H_{29}F_{12}N_9O_4P_2Ru \cdot H_2O$: C 44.15, H 2.44, N 11.53; found (%): C 44.53, H 2.76, N 11.13.

MS analysis of the products of Ru-NBD reacted with GSH and Cys/Hcy

To a solution of 22.3 mg of **Ru-NBD** (0.02 mM) in 10 mL of CH_3CN was added dropwise into 5 mL of aqueous solution of GSH, Cys or Hcy (0.2 mM). After the solution was stirred at 37 ºC for 2 h, the electrospray ionization (ESI) mass spectrum of the solution was measured.

Electrochemical Measurements.

Cyclic voltammetry (CV) was measured on a CHI 660D electrochemical analyzer (Shanghai Chen Hua Instrument Co., Ltd.). Three electrodes were used in electrochemical experiments, including a working electrode (3.0 mm glassy carbon electrode), an auxiliary electrode (0.3 mm platinum wire), and an $Ag/AgNO_3$ (20 mM) electrode reference electrode.

The working electrode was sonicated in 10% HNO_3 for 1 min, followed by polishing with a Al_2O_3 slurry and washing with water for another 1 min before the experiments. The electrochemical experiments were performed in CH_3CN (containing 0.1 M tetrabutylammonium hexafluorophosphate ($TBAPF_6$)).

General procedure for spectrometric analysis

The reaction between **Ru-NBD** and biothiols was performed in a 50 mM Tris-HCl buffer of pH 7.4. The solution of the **Ru-NBD** (10 μM) was incubated with different concentrations of biothiols for 60 min, then the emission spectra of the solutions were measured under steady state and time-gated luminescence modes.

Time-Gated Luminescence Detection

Time-gated emission spectra were performed based on a time-correlated single photon counting (TCSPC) technique using the Edinburgh FS50 spectrofluorometer. Under excitation of 450 nm,

the emission signals from 500 nm to 850 nm were recorded with intervals of 5 nm. The time-gated emission spectra of the mixture were acquired in the absence and presence of biothiols with a delay time of 100 ns.

Detection and discrimination of GSH and Cys/Hcy in vitro

Artificial mixture solution of GSH and Cys is used in proof-of-concept experiments. The GSH and Cys were dissolved in 50 mM Tris-HCl buffer of pH 7.4 with the final concentrations of GSH (8 μM) and Cys (8 μM) as the stock solution. The stock solution was 8-fold diluted with 50 mM Tris-HCl buffer of pH 7.4. **Ru-NBD** (10 μM) was then added into each sample, and the time-gated luminescence spectra were recorded for determination of total concentration of GSH and Cys. Steady state luminescence analysis was then applied to determine the concentration of Cys (λ_{em} = 540 nm). The concentration of GSH was then calculated ($C_{GSH} = C_{total} - C_{Cys}$) All of the analyses were performed three times, and the values are presented as the mean ± SD.

Cell line and cell culture

HeLa cells were purchased from American Type Cell Collection. Cells were cultured in RPMI 1640 media, supplemented with 10% FBS, 1% penicillin, 1% streptomycin sulfate in a humidified 5% CO_2/95% air incubator at 37 ºC. The growth medium was replaced every two days. Cells were routinely detached with trypsin-EDTA solution and then seeded in a 25 mL cell culture bottle. The cells were reached about 80% confluence prior to experiments.

MTT assay of cytotoxicity of Ru-NBD to HeLa cells

The cytotoxicity of **Ru-NBD** to HeLa cells was examined by MTT assay method.[7] HeLa cells were seeded at a density of 5×10^4 cells/mL in a 96-well micro-assay culture plate. After growth at 37 ºC in a 5% CO_2 incubator for 24 h, the culture medium was replaced with the freshly prepared medium containing different concentrations of **Ru-NBD**. The group with the addition of culture medium only was employed as the control, and the wells containing culture media

without cells were used as blanks. After incubation at 37 ºC in a 5% CO_2 incubator for 24 h, cell culture medium was removed, and cells were carefully washed three times with PBS. Then, the MTT solution in PBS (100 μL, 0.5 mg/mL) was added into each well for further incubation for 4 h. The excess MTT solution was then carefully removed from each well, and the formed formazan was dissolved in 100 μL of dimethyl sulfoxide (DMSO). The absorbance at 540 nm was measured in an Infinite M200 Pro Microplate Reader. The results from the five individual experiments were averaged. The following formula was used to calculate the viability of cell growth:

Vialibity (%) = (mean of absorbance value of treatment group-blank)/(mean absorbance value of control-blank) × 100.

Luminescence Imaging of Biothiols in HeLa Cells

For imaging of biothiols in HeLa cells, the cells were seeded in a 20 mm cover glass bottom culture dishes at a density of 5×10^4 cells/mL. After incubation for 24 h at 37°C in a 5% CO_2/95% air incubator, the cell culture medium was removed and then the cells were incubated with **Ru-NBD** (50 μM, containing 0.2% DMSO as the co-solvent) in freshly prepared medium for 5 h. The cells were washed with PBS for three times, and then subjected to the phosphorescence imaging measurements on a microscope. In a control group, HeLa cells were treated with 100 μM NEM for 2 h. The excess NEM was discarded and cells were washed with PBS for three times. Then, the cells were incubated with **Ru-NBD** (50 μM, containing 0.2% DMSO as the co-solvent) in freshly prepared medium for another 5 h. For comparison, HeLa cells were pretreated with GSH (200 μM), Cys (200, 400, and 1600 μM), and Hcy (200, 400, and 1600 μM), respectively. After removal of these biothiols and washing with PBS for three times, the cells were incubated with **Ru-NBD** (50 μM, containing 0.2% DMSO as the co-solvent) in freshly prepared medium for another 5 h.

Time-gated luminescence imaging of biothiols in HeLa cells

HeLa cells were seeded at a density of 5×10^4 cells/mL in a cover glass bottom cell culture dish (ϕ = 22 mm) for time-gated luminescence cell imaging. The cell culture medium was replaced with the freshly prepared medium containing **Ru-NBD** (50 μM, 0.2% DMSO as the co-solvent). The cells were further incubated at 37 °C in a 5% CO_2/95% air incubator for 5 h. The excess **Ru-NBD** was then removed, and the cells were washed with PBS for three times. For comparison, HeLa cells were pretreated with 400 μM Cys and Hcy, respectively for 2 h. Excess Cys and Hcy were removed and washed away, followed by the incubation with **Ru-NBD** (50 μM, containing 0.2% DMSO as the co-solvent) in freshly prepared medium for another 5 h. The cells were then washed three times with PBS (3 × 2 mL/dish) and subjected to time-gated luminescence imaging by Leica SP8 laser-scanning microscope, which is equipped with a pulsed (80 MHz) white light laser. Imaging of both green and red channels at 0-12 ns, 0-4 ns, and 4-12 ns time-gated model were conducted. The intensity analysis of each images was performed by ImagingJ software.

Imaging of biothiols in adult zebrafish

For imaging of biothiols in adult zebrafish, **Ru-NBD** was dissolved with DMSO, and diluted with H_2O (final concentration, 200 μM); biothiols was dissolved directly in water; and NEM was dissolved in DMSO and then diluted with water (final concentration, 500 μM). Four group experiments were performed for imaging of biothiols in adult zebrafish: i) zebrafish was incubated with **Ru-NBD** (200 μM) for 1 h; ii) zebrafish was treated with NEM (500 μM) for 1 h. After washing with water for four times, the zebrafish was then incubated with **Ru-NBD** (200 μM) for another 1 h; iii) zebrafish was treated with NEM (500 μM) for 1 h. After washing with water for four times, the zebrafish was then supplied with Cys (500 μM) for 1 h, followed by the incubation with **Ru-NBD** (200 μM) for another 1 h after four times washing with water; iv) zebrafish was treated with NEM (500 μM) for 1 h. After washing with water for four times, the zebrafish was then supplied with GSH (500 μM) for 1 h, followed by the incubation with

Ru-NBD (200 μM) for another 1 h after four times washing with water.

Visualization and discrimination of biothiols in mouse

The nude mouse (6-8 week old) was anesthetized by isoflurane in a flow of oxygen during all of the experiments. For visualization and discrimination of biothiols in mouse, **Ru-NBD** and NEM were dissolved with DMSO and diluted with physiological saline; biothiols were directly disolved with physiological saline. Five group expeirments were performed: i) the mouse was subcutaneously injected with saline (50 μL); ii) the mouse was subcutaneously injected with **Ru-NBD** (500 μM, 50 μL); iii) the mouse was subcutaneously injected with NEM (1 mM, 50 μL) for 1 h, followed by the injection of **Ru-NBD** (500 μM, 50 μL) at the same area; iv) the mouse was subcutaneously injected with Cys (500 μM, 50 μL) for 1 h, followed by the injection of **Ru-NBD** (500 μM, 50 μL) at the same area; v) the mouse was subcutaneously injected with GSH (500 μM, 50 μL) for 1 h, followed by the injection of **Ru-NBD** (500 μM, 50 μL) at the same area. Under excitation of 480 nm LED, images were recorded at 610 nm and 530 nm emission filters in every 5 min post injection.

2. Theoretical computations of Ru-NBD and Ru-OH

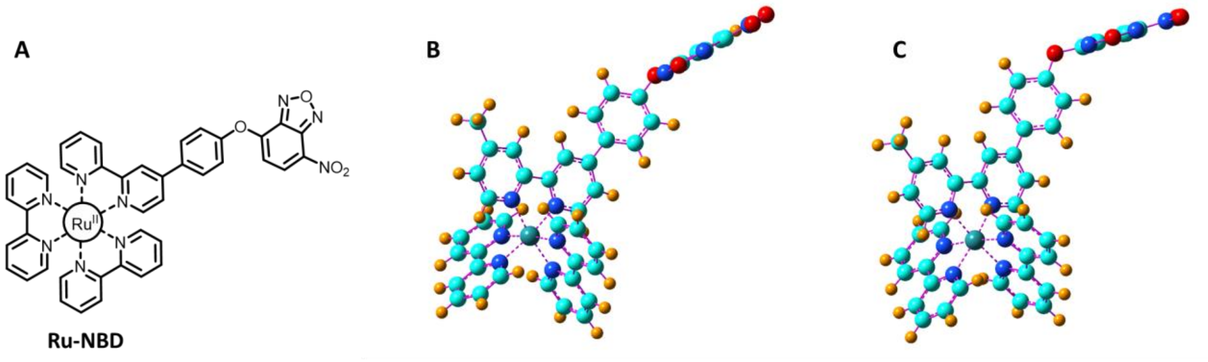

Figure S1. Optimized molecular geometry of **Ru-NBD** (A) in the ground state (S_0) (B) and excited state (T_1) (C) obtained from DFT calculations at B3LYP//6-311+G(d, p)//LANL2DZ level of theory.

Table S1. Cartesian coordinates of **Ru-NBD** in the ground state and excited state

Number	Atom	Coordinates (ground state S_0)			Coordinates (excited state T_1)		
		X	Y	Z	X	Y	Z
**1**	C	3.5374	3.1273	-0.3614	3.2555	3.2051	-0.0879
**2**	C	4.2549	2.0221	-2.3157	3.8669	2.3526	-2.2031
**3**	C	4.4842	3.2341	-2.9651	3.9402	3.6375	-2.7407
**4**	C	4.2295	4.4288	-2.2812	3.6627	4.7320	-1.9160
**5**	C	3.7515	4.3714	-0.9704	3.3169	4.5138	-0.5797
**6**	C	3.0364	2.9578	1.0117	2.9023	2.8583	1.2989
**7**	C	2.4808	1.4057	2.6966	2.5870	1.0931	2.8416
**8**	C	2.1253	2.4269	3.5762	2.2573	1.9894	3.8563
**9**	C	2.2299	3.7562	3.1497	2.2506	3.3589	3.5718
**10**	C	2.6903	4.0202	1.8580	2.5750	3.7955	2.2843
**11**	H	4.4352	1.0770	-2.8082	4.0703	1.4780	-2.8046
**12**	H	4.8540	3.2346	-3.9831	4.2091	3.7674	-3.7811
**13**	H	3.5508	5.2873	-0.4303	3.1006	5.3545	0.0650
**14**	H	2.4179	0.3659	2.9846	2.6070	0.0268	3.0146
**15**	H	1.7747	2.1794	4.5707	2.0117	1.6159	4.8420
**16**	H	2.7763	5.0429	1.5156	2.5696	4.8527	2.0571
**17**	C	0.7455	-1.0268	0.1738	0.8892	-1.2400	0.2483
**18**	C	-0.6215	-1.2256	-0.0409	-0.4626	-1.5481	0.1299
**19**	C	-1.3446	-0.3825	-0.9025	-1.3506	-0.6737	-0.5335
**20**	C	-0.6326	0.6594	-1.5287	-0.7999	0.5146	-1.0638
**21**	C	0.7249	0.8199	-1.2780	0.5516	0.7827	-0.9152
**22**	C	1.5692	-1.8656	1.0617	1.8703	-2.0853	0.9526
**23**	C	1.0884	-2.9871	1.7491	1.5658	-3.3135	1.5454
**24**	C	1.9323	-3.7330	2.5843	2.5600	-4.0559	2.2021
**25**	C	3.2685	-3.3049	2.6975	3.8575	-3.5131	2.2360
**26**	C	3.7024	-2.1878	1.9921	4.1148	-2.2884	1.6292
**27**	H	-1.1355	-2.0204	0.4822	-0.8415	-2.4518	0.5850
**28**	H	-1.1195	1.3321	-2.2239	-1.4089	1.2211	-1.6115
**29**	H	1.2893	1.6093	-1.7536	0.9875	1.6835	-1.3219
**30**	H	0.0554	-3.2916	1.6371	0.5571	-3.7028	1.5018
**31**	H	3.9699	-3.8387	3.3287	4.6661	-4.0385	2.7301
**32**	C	5.5144	-1.4087	-1.3678	5.4831	-1.0127	-1.7203
**33**	C	3.4061	-1.7483	-2.3651	3.3177	-1.5476	-2.4941
**34**	C	3.9417	-2.6460	-3.2873	3.8392	-2.3106	-3.5381
**35**	C	5.3111	-2.9319	-3.2382	5.2268	-2.4250	-3.6647
**36**	C	6.1005	-2.3065	-2.2707	6.0541	-1.7708	-2.7486
**37**	C	6.2570	-0.6905	-0.3200	6.2582	-0.2846	-0.7023
**38**	C	6.1000	0.8226	1.4801	6.1343	1.1031	1.2038
**39**	C	7.4721	0.7479	1.7149	7.5241	1.1583	1.2999
**40**	C	8.2560	-0.0758	0.8987	8.2947	0.4704	0.3577
**41**	C	7.6409	-0.7990	-0.1252	7.6561	-0.2550	-0.6514
**42**	H	2.3547	-1.4981	-2.3685	2.2518	-1.4301	-2.3639
**43**	H	3.2948	-3.1075	-4.0234	3.1658	-2.7994	-4.2304
**44**	H	5.4592	1.4452	2.0882	5.5022	1.6185	1.9122
**45**	H	7.9097	1.3253	2.5201	7.9823	1.7298	2.0968

**46**	N	3.7916	1.9604	-1.0415	3.5317	2.1413	-0.9089
**47**	N	1.4150	0.0007	-0.4450	1.3927	-0.0720	-0.2783
**48**	N	5.4965	0.1213	0.4868	5.5152	0.3950	0.2288
**49**	N	4.1684	-1.1384	-1.4224	4.1200	-0.9115	-1.6072
**50**	N	2.9272	1.6568	1.4397	2.9055	1.5162	1.5938
**51**	N	2.8782	-1.4729	1.1840	3.1442	-1.5833	1.0002
**52**	Ru	3.4476	0.1906	0.0330	3.4207	0.2507	0.0091
**53**	H	8.2357	-1.4370	-0.7655	8.2438	-0.7874	-1.3867
**54**	H	7.1601	-2.5196	-2.2211	7.1286	-1.8542	-2.8371
**55**	H	0.3857	-5.1511	3.1039	1.2098	-5.6678	2.7010
**56**	H	2.0253	-5.8295	3.0789	2.8914	-6.1695	2.4323
**57**	H	1.5243	-4.7933	4.4160	2.4517	-5.3392	3.9252
**58**	H	5.7574	-3.6275	-3.9391	5.6615	-3.0130	-4.4640
**59**	H	9.3256	-0.1542	1.0546	9.3766	0.4964	0.4046
**60**	H	4.3988	5.3871	-2.7580	3.7131	5.7420	-2.3043
**61**	C	-2.7950	-0.5825	-1.1406	-2.7857	-0.9849	-0.6549
**62**	C	-3.6453	0.5237	-1.3493	-3.7425	0.0497	-0.7563
**63**	C	-3.3498	-1.8788	-1.1758	-3.2438	-2.3241	-0.6641
**64**	C	-5.0116	0.3478	-1.5805	-5.1051	-0.2315	-0.8505
**65**	H	-3.2460	1.5314	-1.3171	-3.4314	1.0877	-0.7293
**66**	C	-4.7153	-2.0687	-1.4049	-4.6004	-2.6169	-0.7669
**67**	H	-2.7143	-2.7483	-1.0488	-2.5380	-3.1457	-0.6177
**78**	C	-5.5236	-0.9497	-1.5905	-5.5250	-1.5673	-0.8502
**69**	H	-5.6568	1.2032	-1.7467	-5.8237	0.5761	-0.9167
**70**	H	-5.1459	-3.0622	-1.4423	-4.9535	-3.6412	-0.7838
**71**	O	-6.8953	-1.1876	-1.9087	-6.8696	-1.9465	-0.9662
**72**	C	-7.9394	-0.6007	-1.2474	-7.9023	-1.0049	-0.7887
**73**	C	-9.1321	-0.4480	-1.9259	-8.7346	-0.6832	-1.8538
**74**	C	-7.9011	-0.2208	0.1404	-8.1844	-0.4757	0.4941
**75**	C	-10.2816	0.0830	-1.2915	-9.8326	0.1757	-1.6916
**76**	H	-9.1812	-0.7369	-2.9675	-8.5242	-1.1075	-2.8283
**77**	C	-9.0829	0.3175	0.7965	-9.3173	0.4250	0.6856
**78**	C	-10.2849	0.4689	0.0373	-10.1460	0.7484	-0.4425
**79**	H	-11.1917	0.1959	-1.8665	-10.4619	0.4150	-2.5364
**80**	N	-11.4794	1.0003	0.6198	-11.2371	1.6059	-0.3203
**81**	O	-11.4571	1.3462	1.8452	-11.5246	2.1312	0.8392
**82**	O	-12.5264	1.1119	-0.1011	-11.9653	1.8789	-1.3748
**83**	N	-6.8971	-0.3099	1.0065	-7.5356	-0.6775	1.6462
**84**	N	-8.8141	0.5706	2.0771	-9.3954	0.8028	1.9615
**85**	O	-7.4332	0.1763	2.2219	-8.2750	0.1212	2.6042

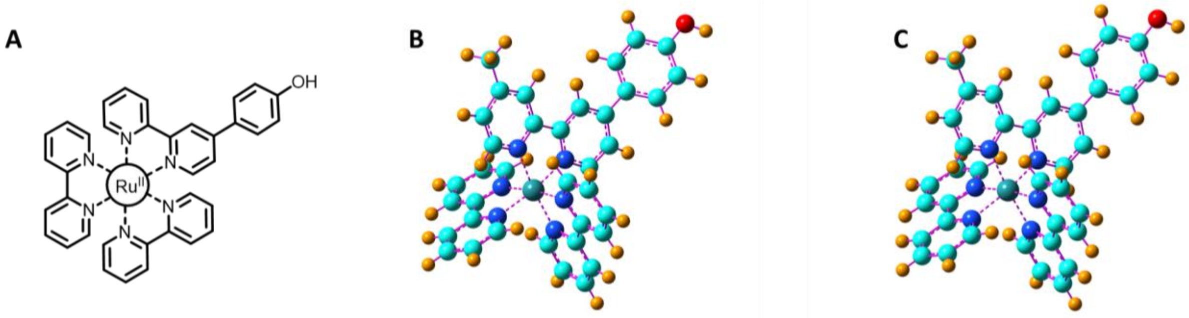

Figure S2. Optimized molecular geometry of **Ru-OH** (A) in the ground state (S_0) (B) and excited state (T_1) (C) obtained from DFT calculations at B3LYP//6-311+G(d, p)//LANL2DZ level of theory.

Table S2. Cartesian coordinates of **Ru-OH** in the ground state and excited state

Number	Atom	Coordinates (ground state S_0)			Coordinates (excited state T_1)		
		X	Y	Z	X	Y	Z
**1**	C	-1.7346	-3.0014	0.1353	-1.7358	-3.0160	0.1096
**2**	C	-2.0408	-2.2358	-2.0494	-1.9789	-2.2443	-2.0809
**3**	C	-2.3799	-3.5076	-2.5007	-2.3423	-3.5090	-2.5369
**4**	C	-2.3946	-4.5610	-1.5858	-2.4032	-4.5577	-1.6205
**5**	C	-2.0677	-4.3027	-0.2568	-2.0958	-4.3080	-0.2839
**6**	C	-1.3706	-2.6327	1.5183	-1.3864	-2.6525	1.5005
**7**	C	-0.7630	-0.8968	2.9575	-0.7651	-0.9182	2.9498
**8**	C	-0.6792	-1.7611	4.0448	-0.7063	-1.7843	4.0368
**9**	C	-0.9530	-3.1149	3.8472	-0.9997	-3.1324	3.8327
**10**	C	-1.3028	-3.5515	2.5717	-1.3432	-3.5694	2.5541
**11**	H	-2.0141	-1.3905	-2.7259	-1.9149	-1.4008	-2.7574
**12**	H	-2.6256	-3.6578	-3.5464	-2.5704	-3.6554	-3.5869
**13**	H	-2.0746	-5.1096	0.4655	-2.1398	-5.1134	0.4384
**14**	H	-0.5613	0.1616	3.0673	-0.5464	0.1373	3.0525
**15**	H	-0.4037	-1.3720	5.0191	-0.4342	-1.4016	5.0141
**16**	H	-1.5186	-4.5992	2.4020	-1.5706	-4.6144	2.3855
**17**	C	1.6062	0.7623	0.2942	1.5779	0.8160	0.2829
**18**	C	2.9998	0.7486	0.2303	2.9745	0.7785	0.2045
**19**	C	3.6866	-0.3225	-0.3640	3.6693	-0.3040	-0.3418
**20**	C	2.8949	-1.3630	-0.8820	2.8508	-1.4095	-0.8258
**21**	C	1.5127	-1.2962	-0.7840	1.4899	-1.3430	-0.7510
**22**	C	0.8302	1.8592	0.9122	0.8220	1.8725	0.8900
**23**	C	1.4137	3.0040	1.4647	1.3757	3.0652	1.4220
**24**	C	0.6244	4.0027	2.0420	0.5725	4.0317	2.0036
**25**	C	-0.7633	3.7984	2.0374	-0.8240	3.7880	2.0453
**26**	C	-1.2881	2.6442	1.4730	-1.3235	2.6154	1.5077
**27**	H	3.5619	1.5628	0.6692	3.5267	1.6092	0.6263
**28**	H	3.3406	-2.2160	-1.3816	3.2985	-2.2798	-1.2885
**29**	H	0.8920	-2.0881	-1.1861	0.8761	-2.1500	-1.1326
**30**	H	2.4903	3.1260	1.4481	2.4471	3.2249	1.3700
**31**	H	-1.4382	4.5314	2.4688	-1.5055	4.5054	2.4908
**32**	C	-2.8509	1.4448	-1.9380	-2.8030	1.4982	-1.9104
**33**	C	-0.6415	1.3078	-2.6832	-0.5724	1.3959	-2.6070
**34**	C	-0.9302	2.0422	-3.8292	-0.8371	2.1763	-3.7283
**35**	C	-2.2369	2.4898	-4.0263	-2.1398	2.6271	-3.9378
**36**	C	-3.2041	2.1864	-3.0711	-3.1303	2.2833	-3.0205
**37**	C	-3.8052	1.0736	-0.8734	-3.7823	1.0818	-0.8848
**38**	C	-4.0854	-0.0165	1.1732	-4.1078	-0.1092	1.0984
**39**	C	-5.4408	0.2961	1.2108	-5.4643	0.2011	1.1202
**40**	C	-5.9886	1.0282	0.1569	-5.9862	0.9852	0.0919
**41**	C	-5.1609	1.4201	-0.8926	-5.1366	1.4289	-0.9193
**42**	H	0.3578	0.9366	-2.4923	0.4214	1.0174	-2.4048

**43**	H	-0.1421	2.2524	-4.5442	-0.0329	2.4172	-4.4146
**44**	H	-3.6181	-0.5781	1.9728	-3.6613	-0.7110	1.8806
**45**	H	-6.0435	-0.0305	2.0513	-6.0861	-0.1673	1.9285
**46**	N	-1.7251	-1.9784	-0.7655	-1.6842	-1.9989	-0.7926
**47**	N	0.8643	-0.2631	-0.2121	0.8075	-0.2721	-0.2306
**48**	N	-3.2795	0.3580	0.1610	-3.2838	0.3197	0.1254
**49**	N	-1.5717	1.0105	-1.7555	-1.5279	1.0620	-1.7194
**50**	N	-1.0993	-1.3122	1.7210	-1.0964	-1.3384	1.7145
**51**	N	-0.5196	1.6858	0.9184	-0.5414	1.6718	0.9458
**52**	Ru	-1.2231	-0.0845	0.0111	-1.1766	-0.1044	0.0178
**53**	H	-5.5712	1.9906	-1.7168	-5.5296	2.0385	-1.7233
**54**	H	-4.2230	2.5257	-3.2104	-4.1464	2.6249	-3.1719
**55**	H	2.3198	5.2473	2.5419	2.2312	5.3444	2.4565
**56**	H	0.8336	6.1420	2.1606	0.7094	6.1813	2.0837
**57**	H	0.9848	5.3135	3.7107	0.9119	5.3862	3.6447
**58**	H	-2.5020	3.0649	-4.9078	-2.3857	3.2364	-4.8015
**59**	H	-7.0412	1.2923	0.1497	-7.0385	1.2500	0.0736
**60**	H	-2.6549	-5.5672	-1.8983	-2.6847	-5.5570	-1.9364
**61**	C	5.1630	-0.3521	-0.4342	5.1256	-0.3471	-0.4142
**62**	C	5.8648	-1.5710	-0.3831	5.8207	-1.5704	-0.5960
**63**	C	5.9132	0.8352	-0.5512	5.9164	0.8285	-0.3035
**64**	C	7.2566	-1.6088	-0.4409	7.2095	-1.6236	-0.6456
**65**	H	5.3252	-2.5065	-0.2686	5.2736	-2.5038	-0.6716
**66**	C	7.3028	0.8100	-0.6173	7.3004	0.7861	-0.3566
**67**	H	5.4079	1.7942	-0.6204	5.4381	1.7970	-0.2001
**68**	C	7.9806	-0.4151	-0.5593	7.9593	-0.4449	-0.5234
**69**	H	7.7772	-2.5615	-0.3837	7.7119	-2.5794	-0.7736
**70**	H	7.8709	1.7295	-0.7212	7.8874	1.6966	-0.2805
**71**	O	9.3449	-0.3813	-0.6230	9.3223	-0.4250	-0.5643
**72**	H	9.7103	-1.2813	-0.5808	9.6738	-1.3245	-0.6786

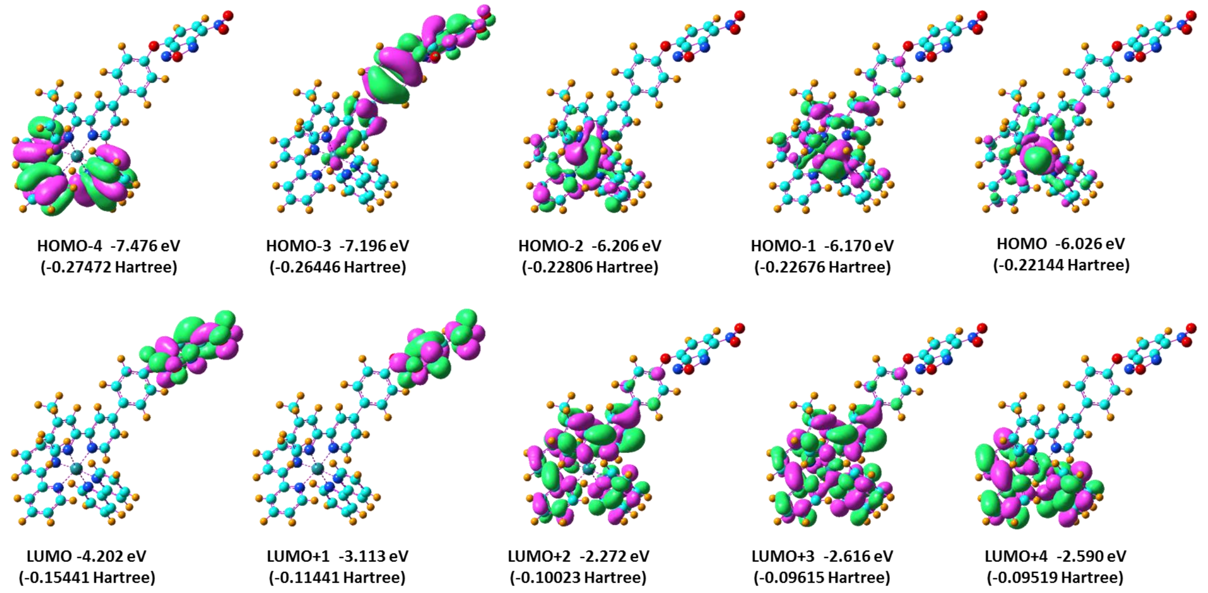

Figure S3. Representative frontier molecular orbital distributions of **Ru-NBD** and their corresponding energy at ground state (S_0) optimized geometries (isodensity contour = 0.02 a.u.).

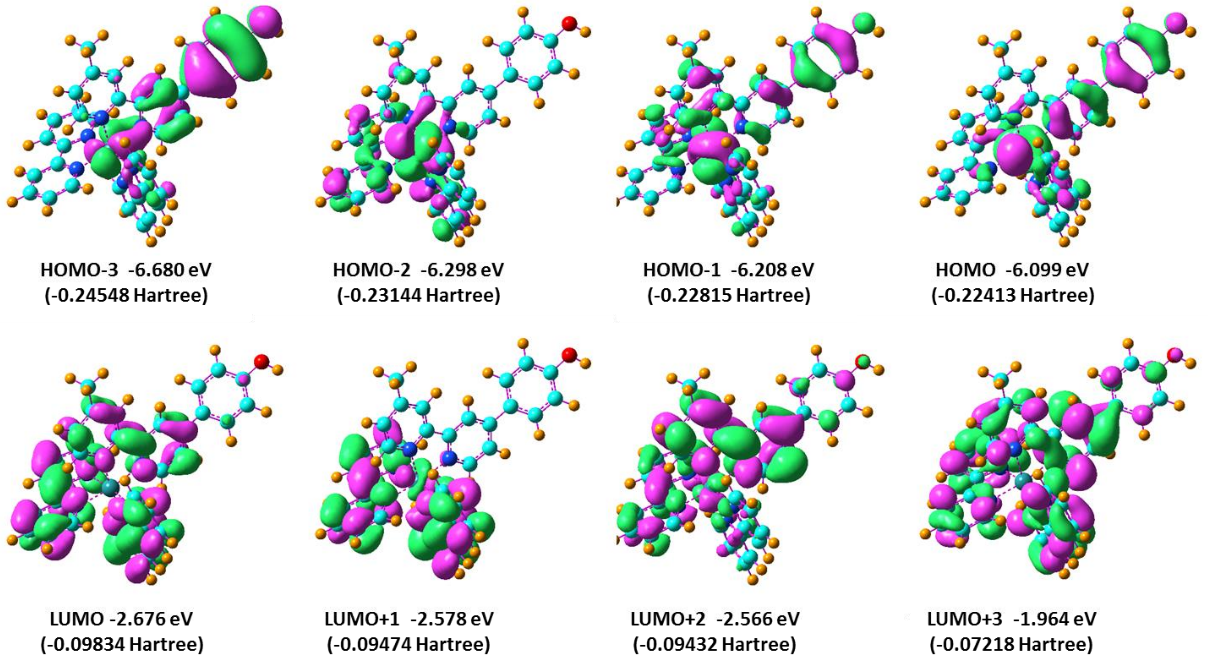

Figure S4. Representative frontier molecular orbital distributions of **Ru-OH** and their corresponding energy at ground state (S_0) optimized geometries (isodensity contour = 0.02 a.u.).

Table S3. Absorptions of **Ru-NBD** and **Ru-OH** in aqueous solution from the calculation conducted by TDDFT//B3LYP//6-311+G(d, p), based on the optimized molecular geometries in the ground state.

Complexes	State	Energy (eV) [a]	Wavelength (nm)	*f* [b]	Composition [c]	Contribution [d] (%)	Assignment [e]
**Ru-NBD**	S_1	1.5905	779.55	0.0063	HOMO→LUMO	99.13	ET
	S_2	1.7244	718.99	0.0127	HOMO-1→LUMO	97.99	ET
	S_3	1.7797	696.66	0.0003	HOMO-2→LUMO	98.91	ET
	S_4	2.4698	502.00	0.0116	HOMO→LUMO+2	63.22	^{1}MLCT, ^{1}ML'CT
					HOMO→LUMO+3	37.72	^{1}MLCT, ^{1}ML'CT
	S_5	2.4827	499.40	0.0014	HOMO→LUMO+2	33.16	^{1}MLCT, ^{1}ML'CT
					HOMO→LUMO+3	49.66	^{1}MLCT, ^{1}ML'CT
					HOMO→LUMO+4	14.93	^{1}MLCT, ^{1}ML'CT
	S_6	2.4854	498.84	0.0002	HOMO→LUMO+3	15.36	^{1}MLCT, ^{1}ML'CT
					HOMO→LUMO+4	80.79	^{1}MLCT
	S_7	2.5144	493.10	0.1721	HOMO-3→LUMO	92.76	ET, ^{1}IL'CT
	S_8	2.6567	466.68	0.0008	HOMO-2→LUMO+3	43.61	^{1}MLCT, ^{1}ML'CT
					HOMO-1→LUMO+4	50.45	^{1}MLCT
	S_9	2.6787	462.86	0.0414	HOMO-1→LUMO+2	74.17	^{1}MLCT, ^{1}ML'CT
					HOMO-1→LUMO+3	17.57	^{1}MLCT, ^{1}ML'CT

	S_{10}	2.6887	461.13	0.0010	HOMO-2→LUMO+2	98.43	ET
**Ru-OH**	S_1	2.6116	474.74	0.0021	HOMO→LUMO	70.36	^{1}ML'CT, ^{1}MLCT, ^{1}IL'CT
					HOMO→LUMO+2	17.26	^{1}ML'CT, ^{1}MLCT, ^{1}IL'CT
	S_2	2.6180	473.58	0.0001	HOMO-1→LUMO+1	11.04	^{1}MLCT, ^{1}L'LCT
					HOMO→LUMO+1	84.61	^{1}MLCT, ^{1}L'LCT
	S_3	2.6687	464.58	0.0099	HOMO-1→LUMO+2	10.55	^{1}ML'CT, ^{1}MLCT, ^{1}IL'CT
					HOMO→LUMO	18.34	^{1}ML'CT, ^{1}MLCT, ^{1}IL'CT
					HOMO→LUMO+2	65.67	^{1}ML'CT, ^{1}MLCT, ^{1}IL'CT
	S_4	2.7726	447.17	0.0111	HOMO-1→LUMO	43.00	^{1}ML'CT, ^{1}MLCT, ^{1}IL'CT
					HOMO-1→LUMO+1	30.82	^{1}MLCT, ^{1}IL'CT
	S_5	2.7833	445.46	0.0214	HOMO-2→LUMO	10.19	^{1}ML'CT, ^{1}MLCT
					HOMO-2→LUMO+1	13.33	^{1}MLCT
					HOMO-1→LUMO	13.19	^{1}ML'CT, ^{1}MLCT, ^{1}IL'CT
					HOMO-1→LUMO+1	24.08	^{1}MLCT, ^{1}IL'CT
	S_6	2.8589	433.68	0.0296	HOMO-2→LUMO	68.74	^{1}MLCT, 1ML'CT
					HOMO-2→LUMO+2	17.77	^{1}MLCT, 1ML'CT
	S_7	2.9109	425.92	0.2253	HOMO-2→LUMO	12.99	^{1}MLCT, 1ML'CT
					HOMO-2→LUMO+1	29.57	^{1}MLCT
					HOMO-1→LUMO+2	37.31	^{1}ML'CT, ^{1}MLCT, ^{1}IL'CT
	S_8	2.9196	424.65	0.0950	HOMO-2→LUMO+2	55.44	^{1}MLCT, 1ML'CT
					HOMO-1→LUMO+1	18.95	^{1}MLCT, ^{1}IL'CT
	S_9	3.1313	395.95	0.0009	HOMO-2→LUMO+1	41.33	^{1}MLCT
					HOMO-2→LUMO+2	10.21	^{1}MLCT, 1ML'CT
					HOMO-1→LUMO+2	25.45	^{1}ML'CT, ^{1}MLCT, ^{1}IL'CT
	S_{10}	3.4208	362.44	0.0280	HOMO→LUMO+3	87.54	^{1}ML'CT, ^{1}MLCT

[a]Only the selected low-lying excited states are presented. [b]Oscillator strength. [c]Only the main configurations are presented. [d]Contributions over 10% are presented. [e]MLCT: metal to ligand charge transfer; LLCT: ligand to ligand charge transfer; ILCT: intra-ligand charge transfer (L: bpy; L': NBD-bpy or OH-bpy).

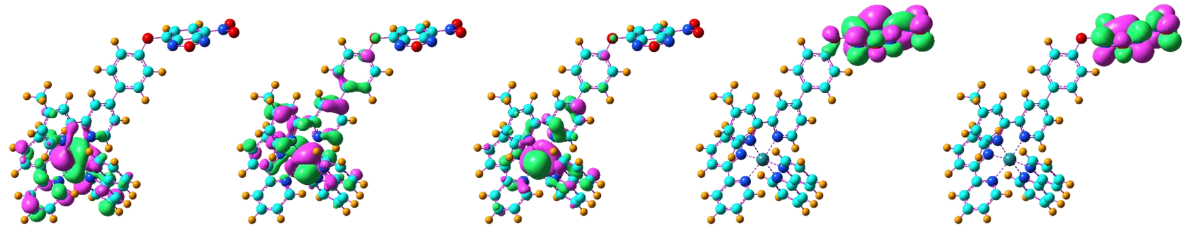

Figure S5. Representative frontier molecular orbital distributions of **Ru-NBD** and their corresponding energy at excited state (T_1) optimized geometries (isodensity contour = 0.02 a.u.).

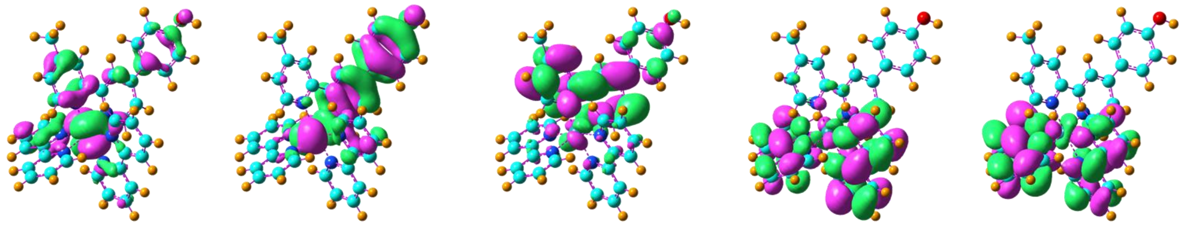

Figure S6. Representative frontier molecular orbital distributions of **Ru-OH** and their corresponding energy at excited state (T_1) optimized geometries (isodensity contour = 0.02 a.u.).

Table S4. Selected electronic excitation energies (eV) and corresponding oscillator strengths (f), main configurations and contributions of the low-lying electronically excited states of **Ru-NBD** and **Ru-OH** in aqueous solution from the calculation conducted by TDDFT//B3LYP//6-311+G(d, p), based on the optimized molecular geometries in the first excited triplet states of the complexes.

Complexes	State	Energy (eV) [a]	Wavelength (nm)	*f* [b]	Composition [c]	Contribution [d] (%)	Assignment [e]
**Ru-NBD**	T_1	1.0277	1206.37	0.0000	HOMO→LUMO	97.36	ET
	T_2	1.2333	1005.33	0.0000	HOMO-1→LUMO	95.07	ET
	T_3	1.3259	935.08	0.0000	HOMO-2→LUMO	62.82	ET
**Ru-OH**	T_1	1.8738	661.69	0.0000	HOMO→LUMO	86.35	^{3}ML'CT, ^{3}IL'CT
	T_2	2.2793	543.95	0.0000	HOMO-1→LUMO	82.38	^{3}ML'CT
	T_3	2.3771	521.58	0.0000	HOMO-1→LUMO+1	43.03	^{3}MLCT
					HOMO-1→LUMO+2	38.49	^{3}MLCT

[a]Only the selected low-lying excited states are presented. [b]Oscillator strength. [c]Only the main configurations are presented. [d]Contributions over 10% are presented. [e]MLCT: metal to ligand charge transfer; LLCT: ligand to ligand charge transfer; ILCT: intra-ligand charge transfer (L: ppy; L': NBD-bpy or OH-bpy).

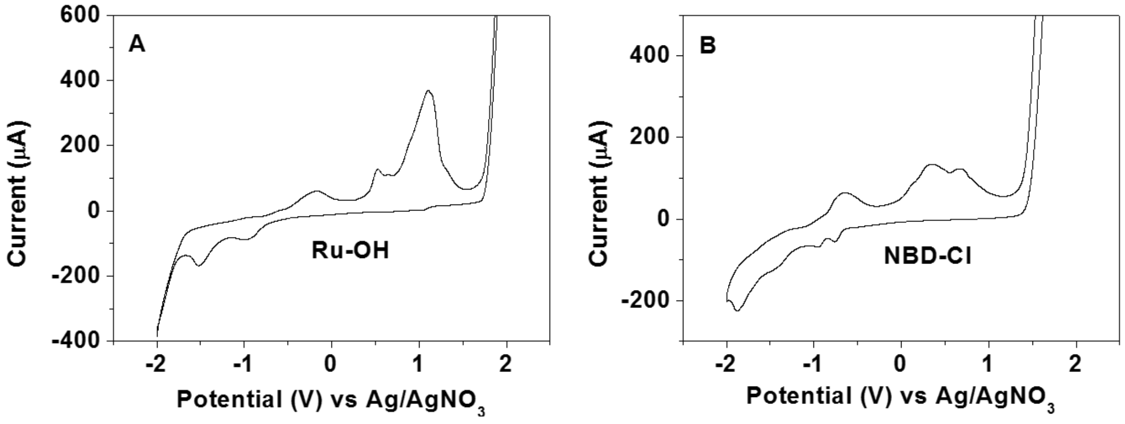

Figure S7. Cyclic voltammograms of 1.0 mM **Ru-OH** (A) and NBD-Cl (B) in CH_3CN containing 0.1 M $TBAPF_6$. Scan rate: 0.1 V/s. Working electrode: platinum; counter electrode: platinum wire; reference electrode: $Ag/AgNO_3$.

3. Characterizations of Ru-NBD

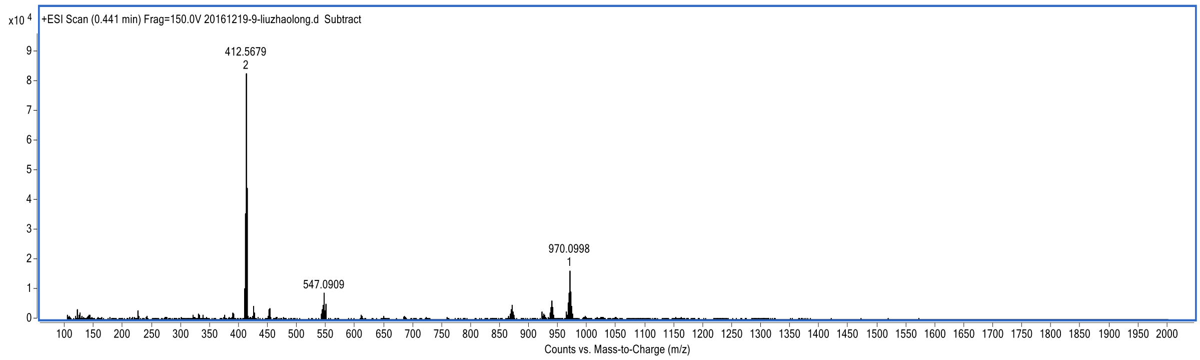

Figure S8. ESI-HRMS of **Ru-NBD**.

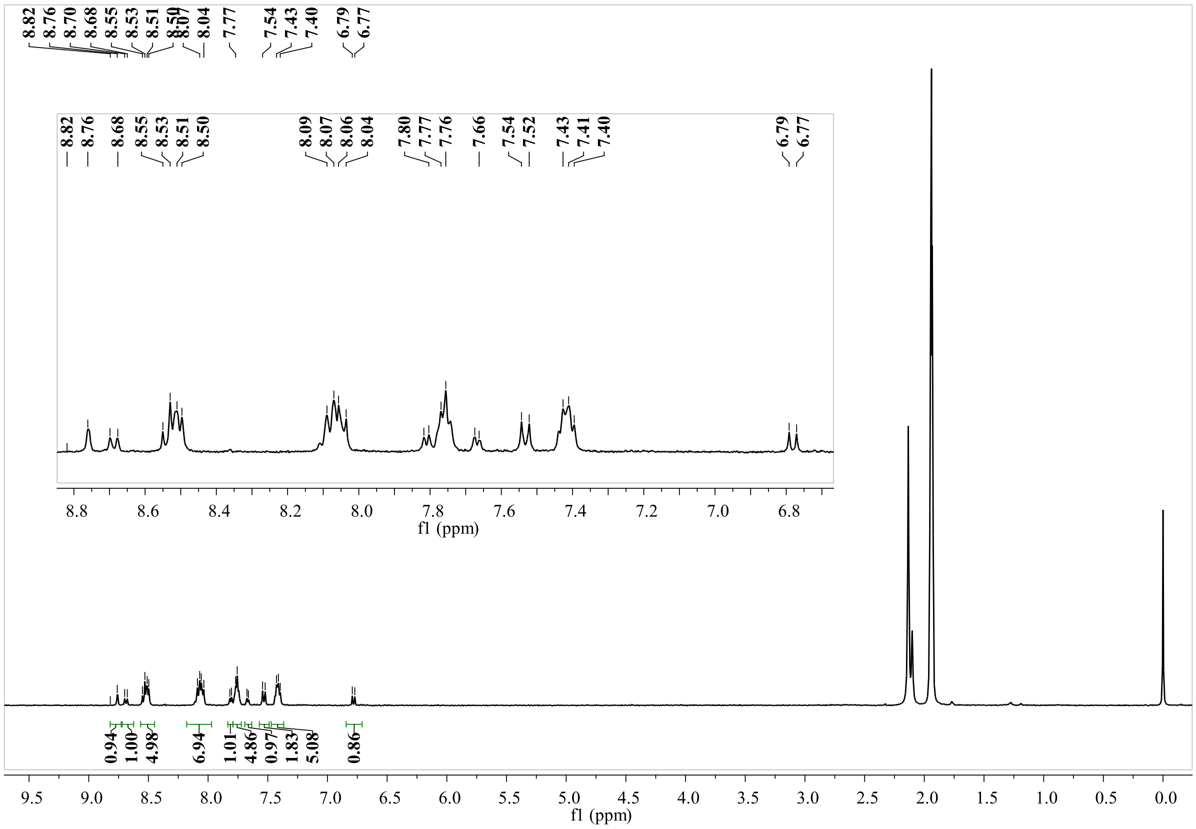

Figure S9. ^{1}H NMR spectrum of **Ru-NBD**.

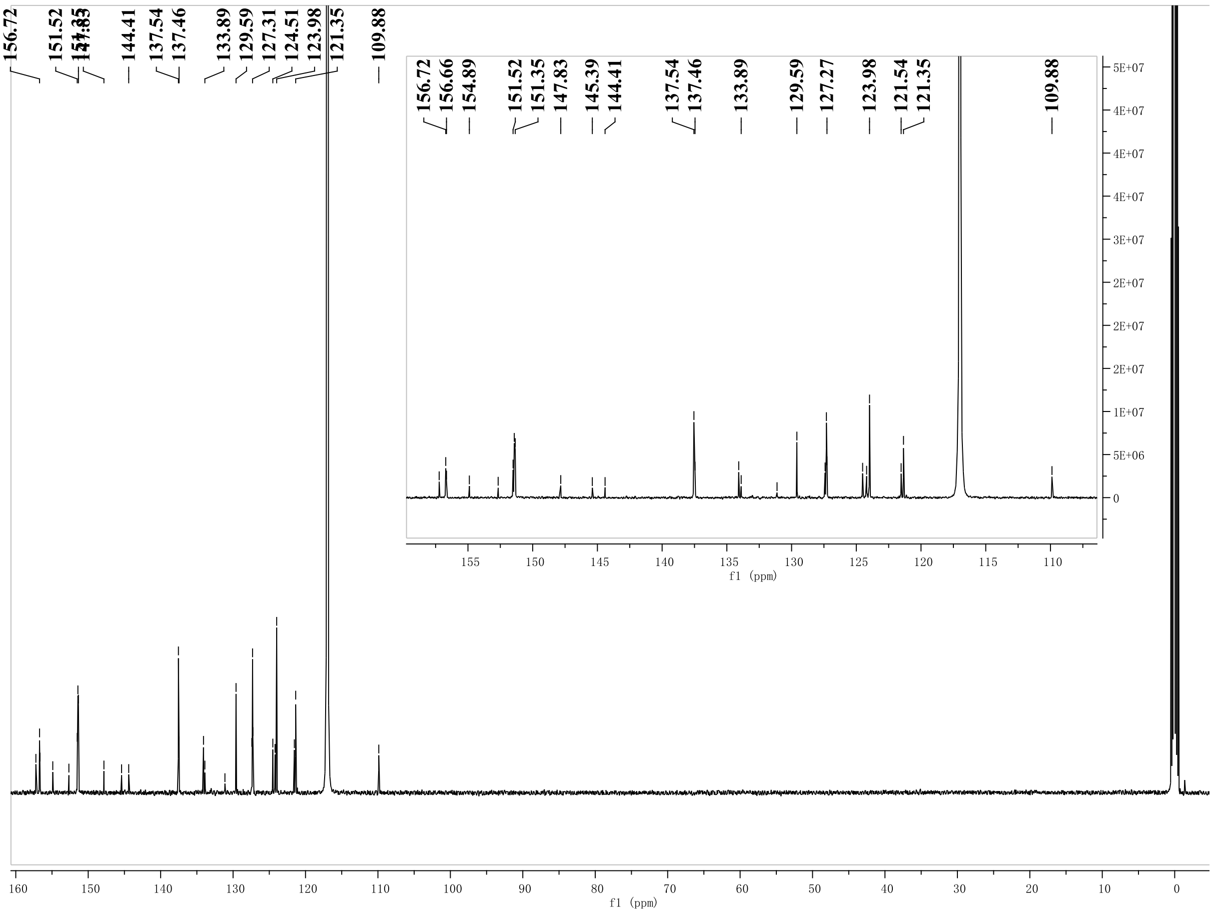

Figure S10. ^{13}C NMR spectrum of **Ru-NBD**.

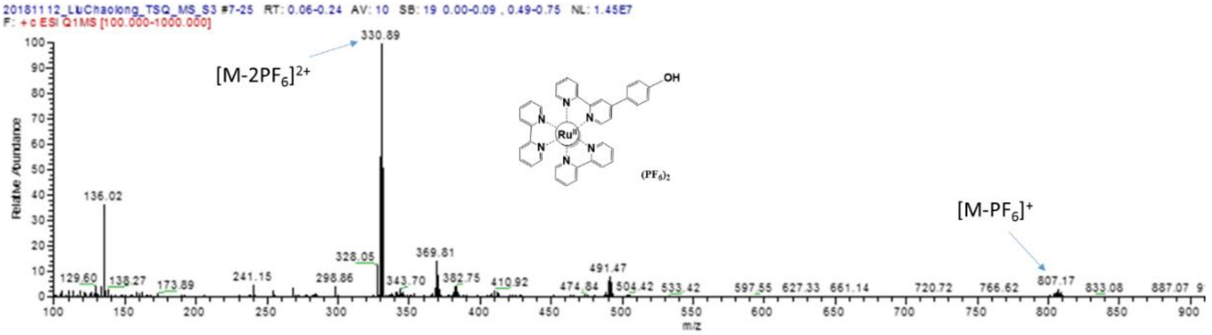

Figure S11. MS of the product **Ru-OH** after **Ru-NBD** (20 μM) reacted with Cys (200 μM).

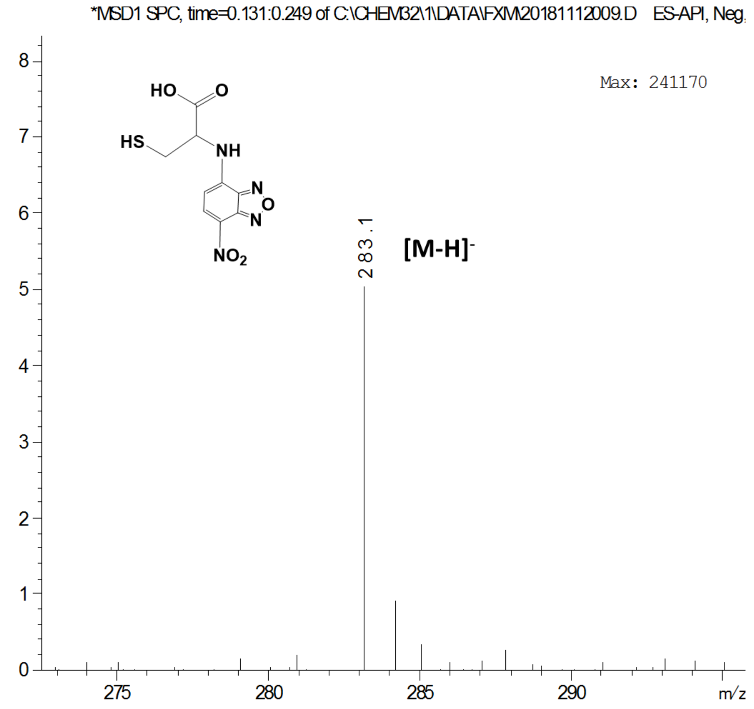

Figure S12. MS of **NBD-NR1**.

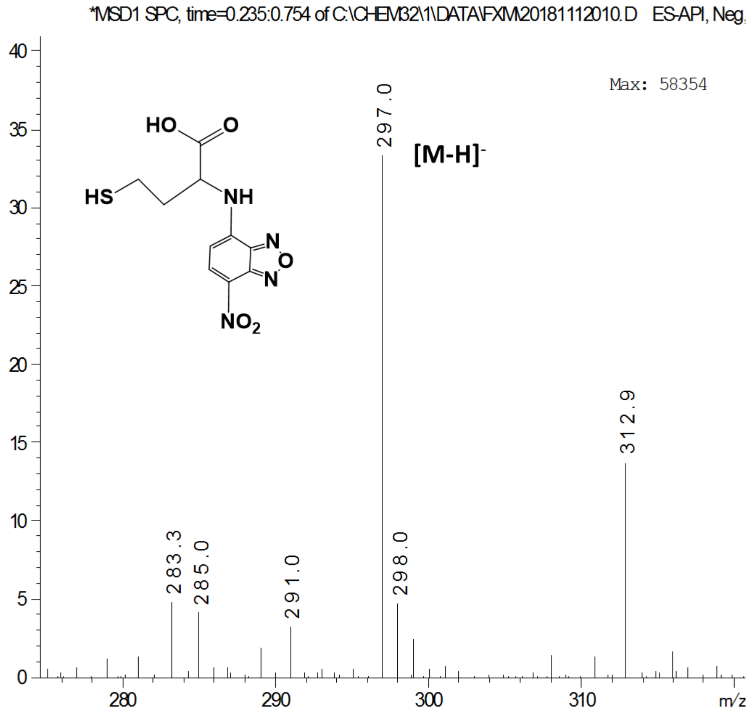

Figure S13. MS of **NBD-NR2**.

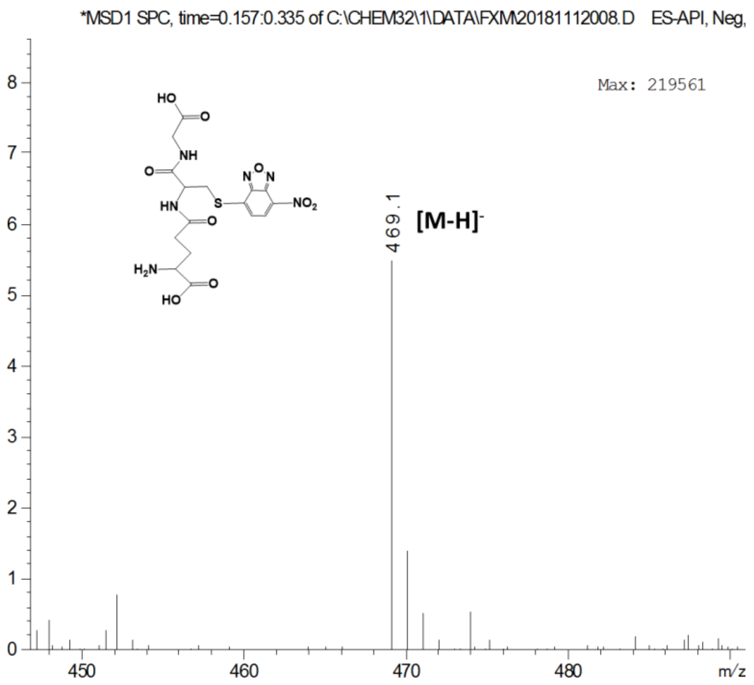

Figure S14. MS of **NBD-SR**.

4. Luminescence spectra of Ru-NBD for biothiols

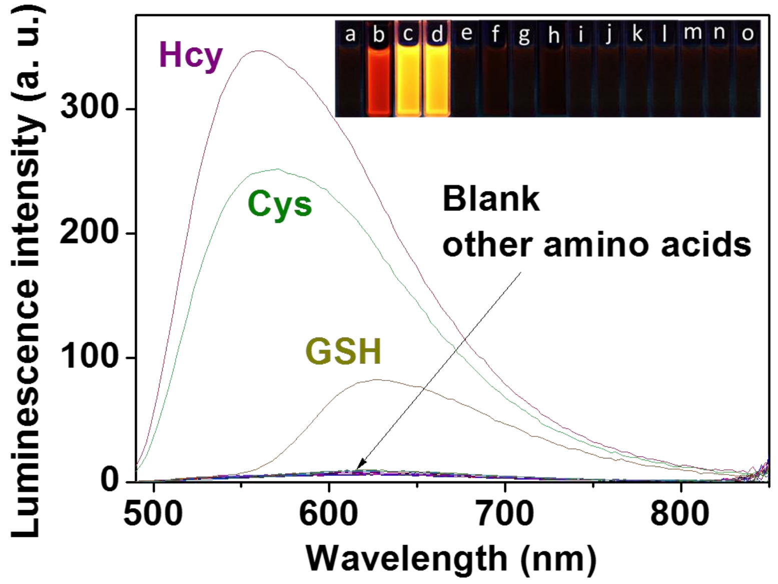

Figure S15. Luminescence intensities of **Ru-NBD** (10 μM) upon reaction with various amino acids (100 μM) in 50 mM Tris-HCl buffer of pH 7.4. Inset: luminescence color photographs of the products of **Ru-NBD** reacted with different amino acids under a 365 nm UV lamp. Amino acids: (a) blank, (b) GSH, (c) Cys, (d) Hcy, (e) tryptophan, (f) threonine, (g) glycine, (h) valine, (i) leucine, (j) histidine, (k) proline, (l) serine, (m) tyrosine, (n) alanine, and (o) aspartic acid.

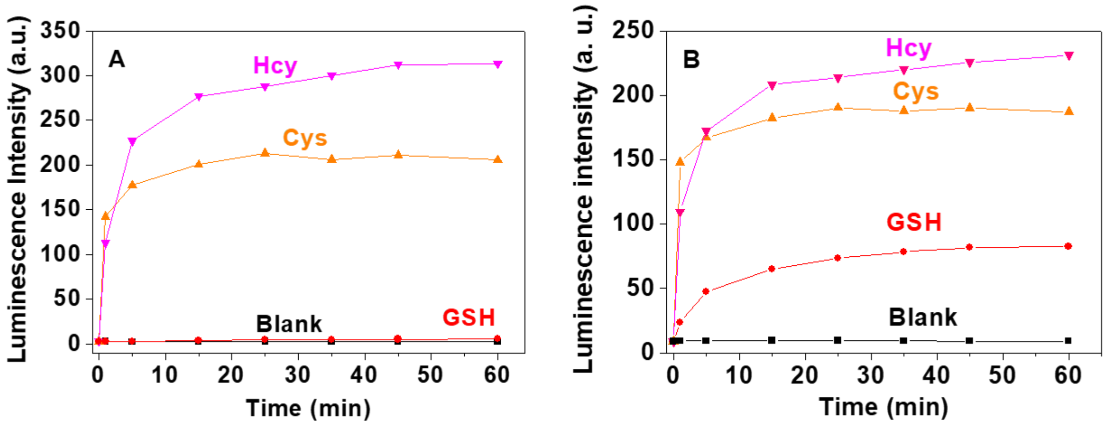

Figure S16. Time courses luminescence response of **Ru-NBD** (10 μM) to different biothiols (200 μM) in 50 mM Tris-HCl buffer (pH 7.4). λ_{ex} = 480 nm; λ_{em} = 540 nm (A); λ_{em} = 628 nm (B).

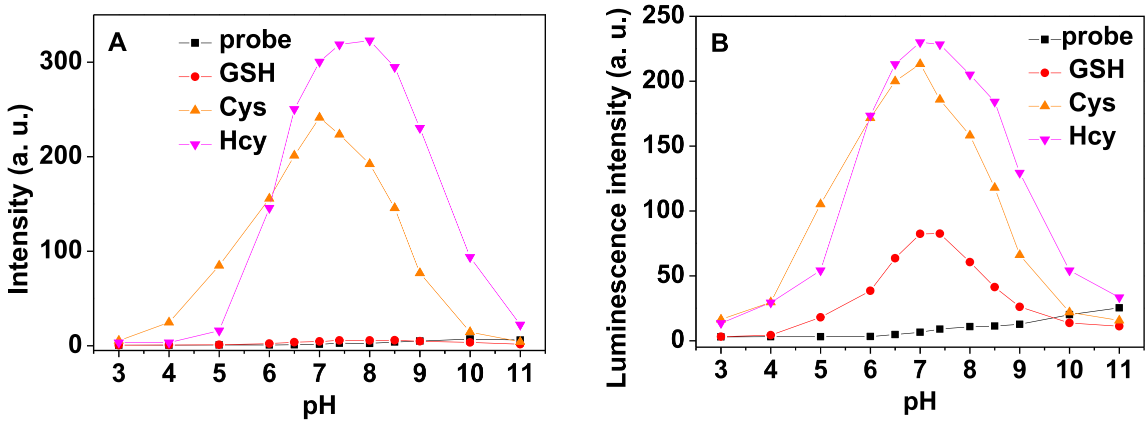

Figure S17. Luminescence intensity changes of **Ru-NBD** (10 μM) in Tris-HCl buffer with different pH in the absence and presence of 20 equiv. of GSH, Cys and Hcy. λ_{ex} = 480 nm; λ_{em} = 540 nm (A); λ_{em} = 628 nm (B).

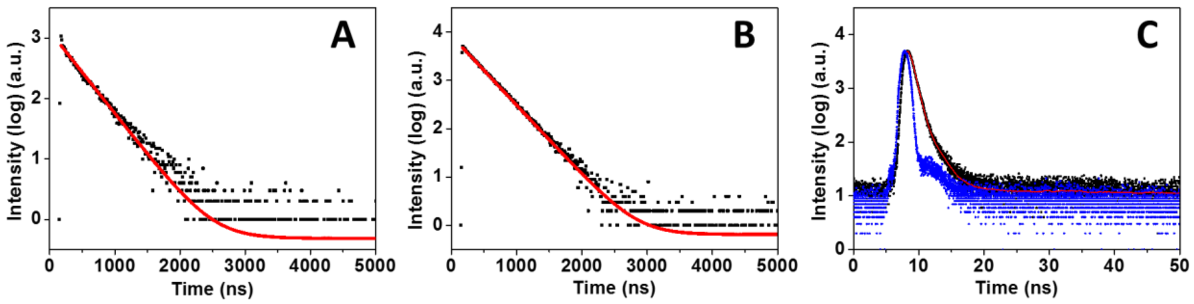

Figure S18. The luminescence decay traces of **Ru-NBD** (A), **Ru-OH** (B) and **NBD-NR1**(C).

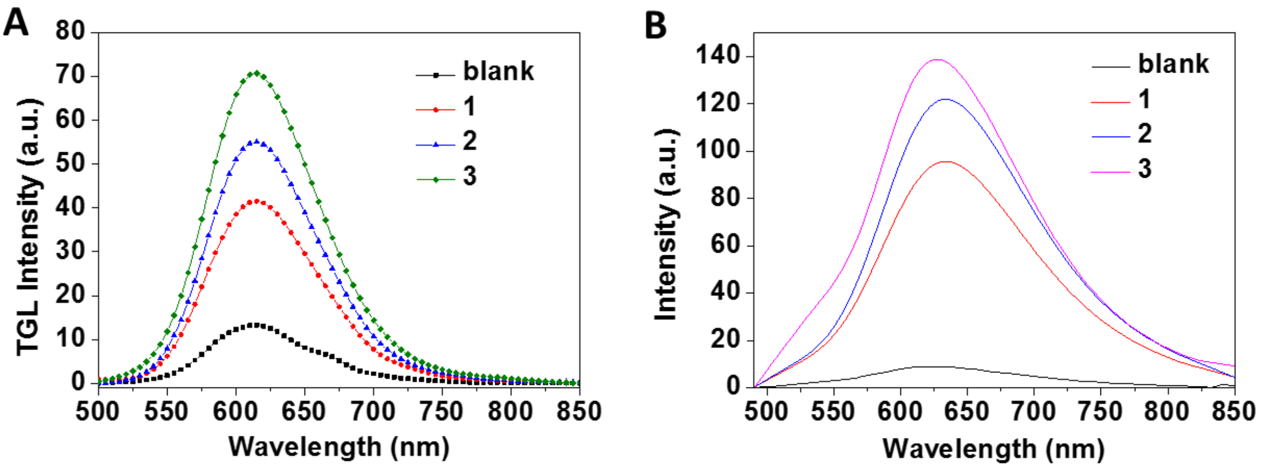

Figure S19. Time-gated (A, delay time: 100 ns) and steady-state (B, delay time: 0 ns) emission spectra. Mixture was 8-foled diluted for Time-gated luminescence analysis. Excitation was performed at 450 nm.

Table S5. Detection of biothoils using **Ru-NBD** probe under phosphorescence and time-gated luminescence assay model.

Sample number	Total biothiols	Found (μ M) (n=3)	Recovery biothiols (%)	Added Cys (μ M)	Found Cys (μ M) (n=3)	Recovery Cys (%)	Added GSH (μ M)	Found (μ M) (n=3)	Recovery GSH (%)
1 (mixture)	16	16.56±0.53	103.50	-	9.05±0.23	113.12	-	7.51±0.31	85.86
2 (mixture+GSH)	22	21.52±0.56	97.82	-	9.02±0.16	112.75	6	12.50±0.21	89.29
3 (mixture+GSH+Cys)	28	30.08±0.33	107.43	6	15.37±0.15	109.78	6	14.71±0.19	105.07

5. Detection and discrimination of biothiols in live cells and organisms

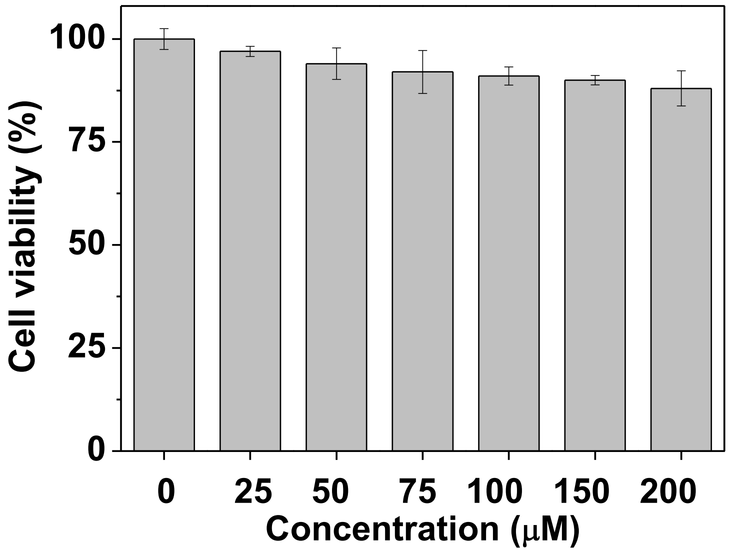

Figure S20. Viabilities of Hela cells incubated with **Ru-NBD** at increasing concentrations (0, 25, 50, 75, 100, 150 and 200 μM) for 24 h at 37 ºC.

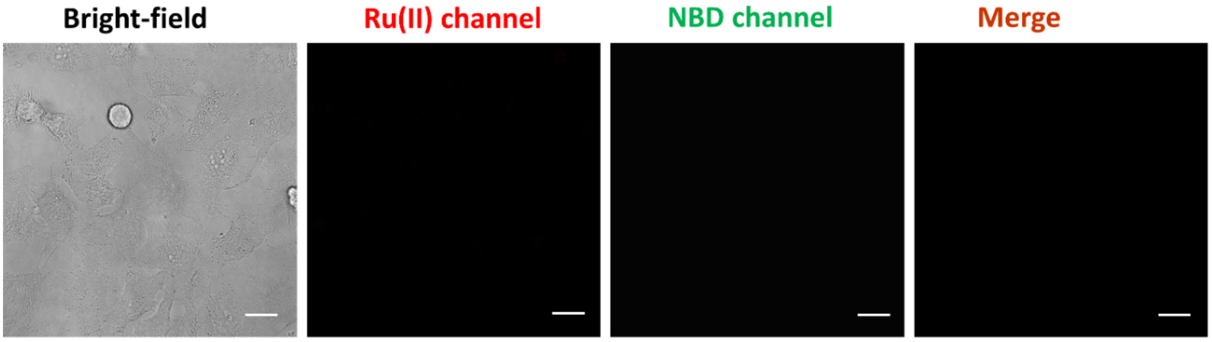

Figure S21. Bright field and luminescence imaging of HeLa cells. Scale bar: 20 μm.

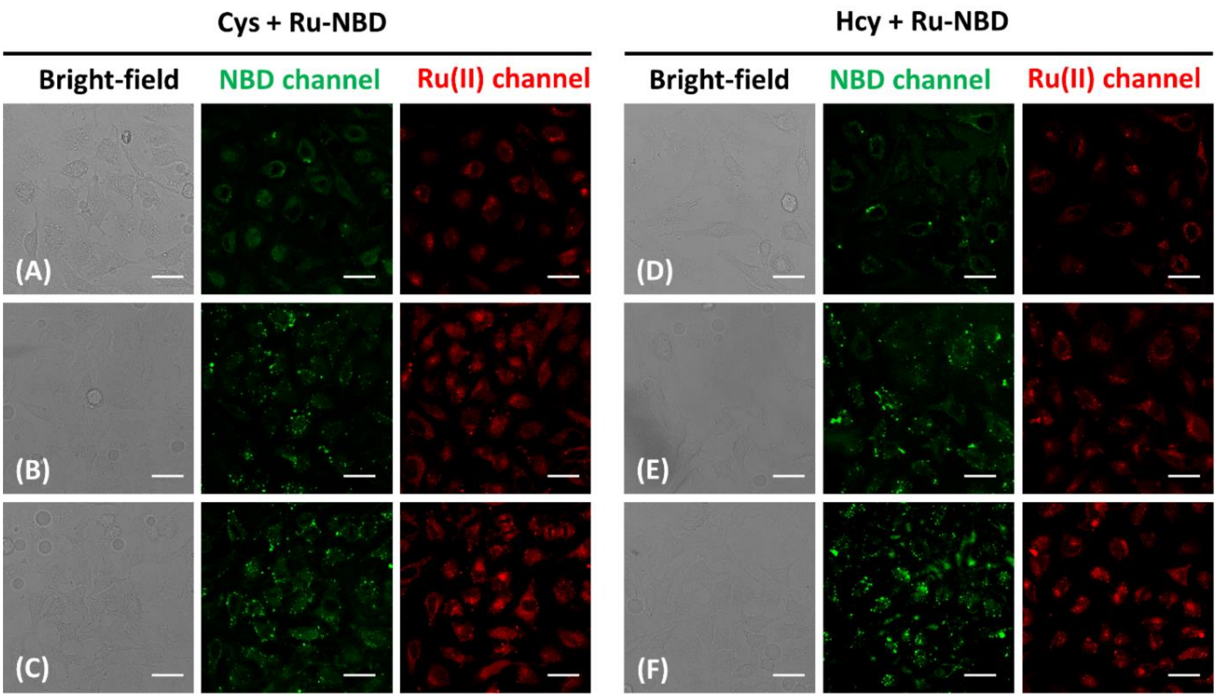

Figure S22. Luminescence imaging of exogenous and endogenous Cys (A-C) and Hcy (D-F) in HeLa cells. HeLa cells were incubated 200 (A, D), 400 (B, E) and 1600 μM (C, F) Cys and Hcy, respectively, and then treated with **Ru-NBD** (50 μM).

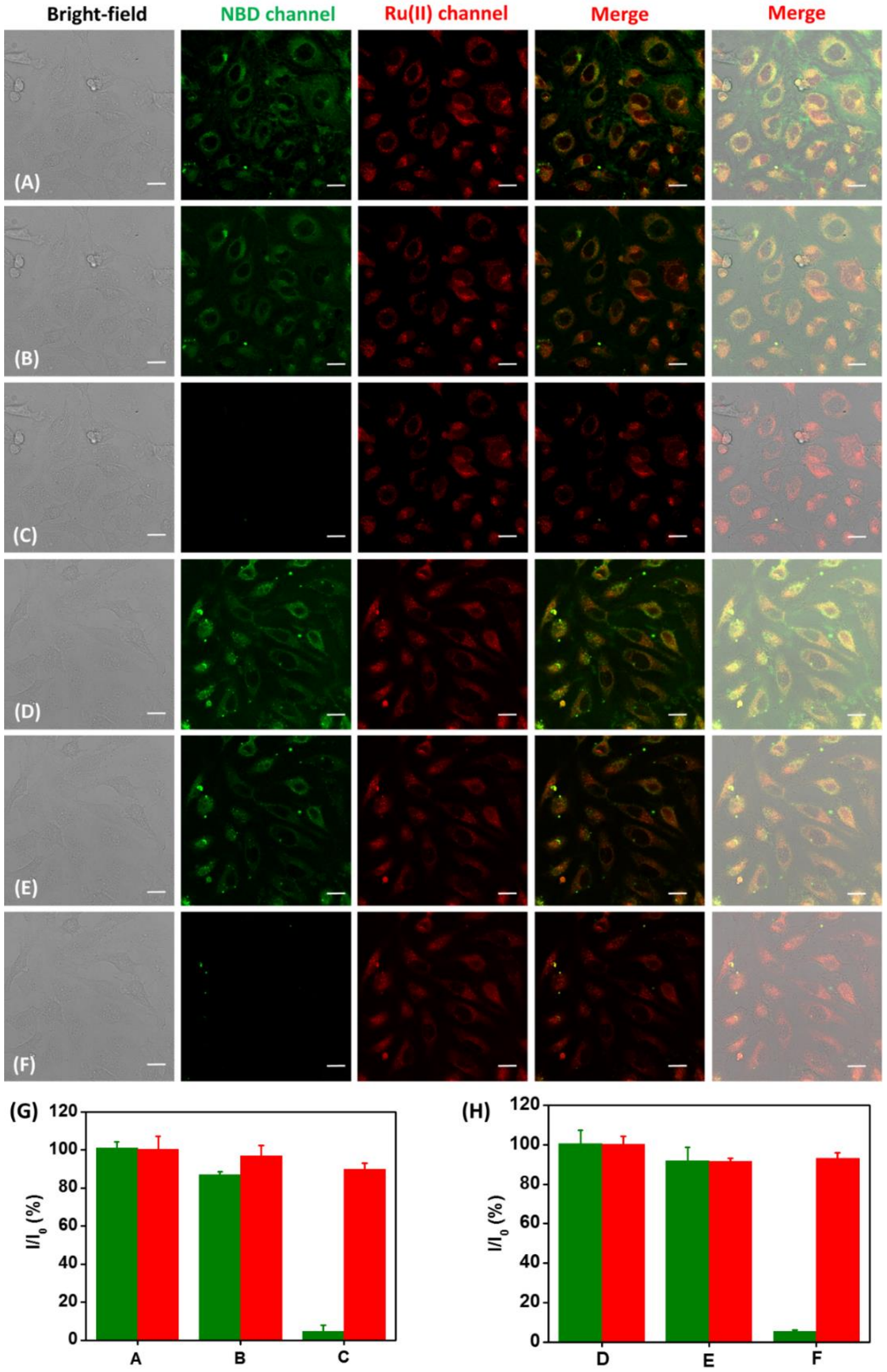

Figure S23. Time-gated luminescence imaging of HeLa cells treated with 400 μM Cys (A-C) and Hcy (D-F) and 50 μM **Ru-NBD**. The signals from green NBD channel were collected within 0-12 ns (A, D), 0-4 ns (B, E), and 4-12 ns (C, F). The signals from red Ru(II) channel were collected under normal condition (inactive time-gated luminescence imaging). Changes of intracellular red and green signals of group A-C (G) and D-F (H). Scale bar: 20 μm.

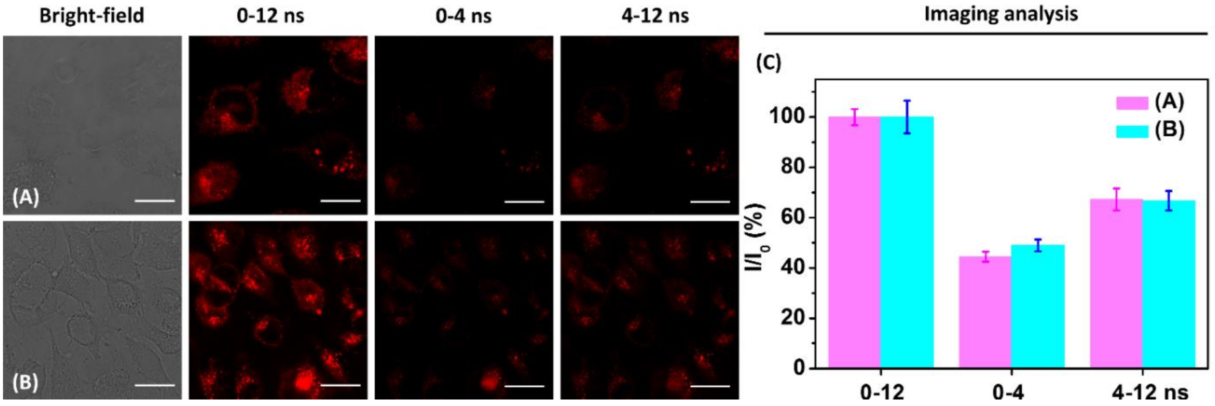

Figure S24. Time-gated luminescence imaging of HeLa cells treated with 400 µM Cys (A) and Hcy (B) and 50 µM **Ru-NBD**. The signals from red Ru(II) channel were collected within 0-12 ns (A, D), 0-4 ns (B, E), and 4-12 ns (D, F). Changes of intracellular red and green signals (C). Scale bar: 20 µm.

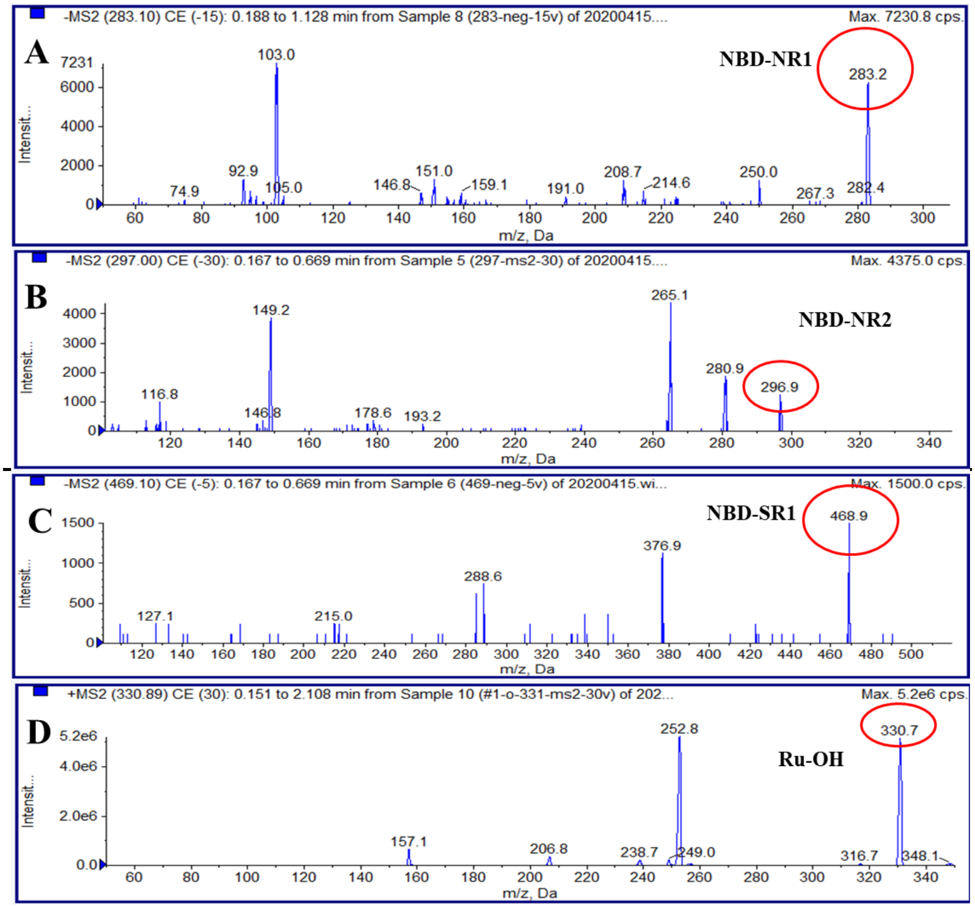

Figure S25. MS of **NBD-NR1** (A), **NBD-NR2** (B)**, NBD-SR1** (C)**, and Ru-OH** (D) in cell lysate.

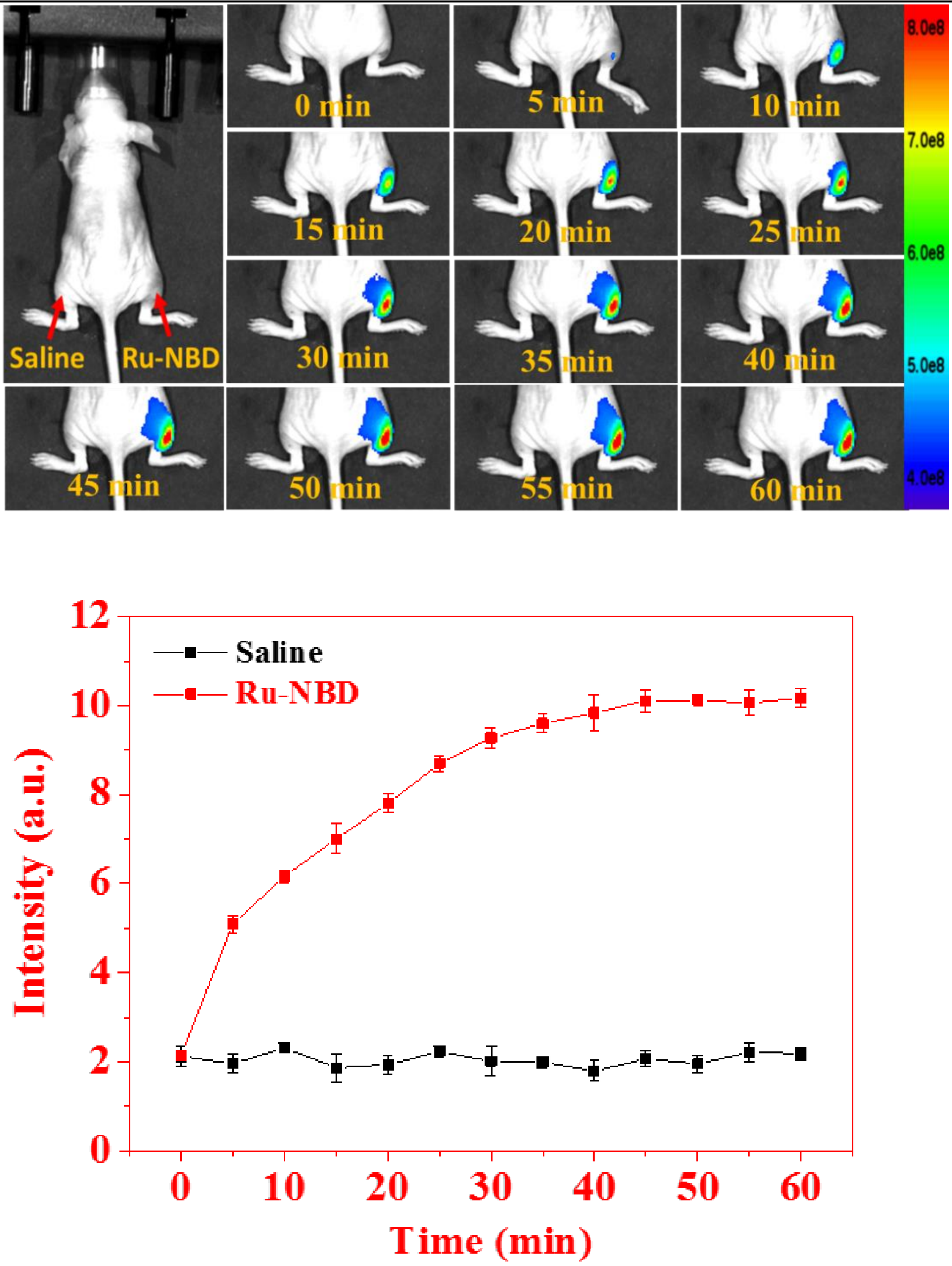

Figure S26. Luminescence imaging of biothiols in live mice using **Ru-NBD** as a probe. Ex: 465nm, Em: 610 nm.

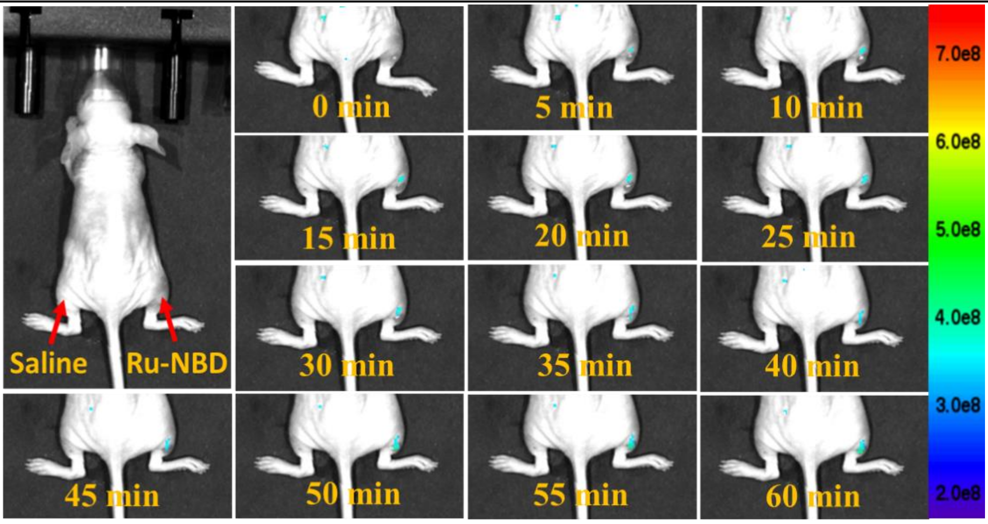

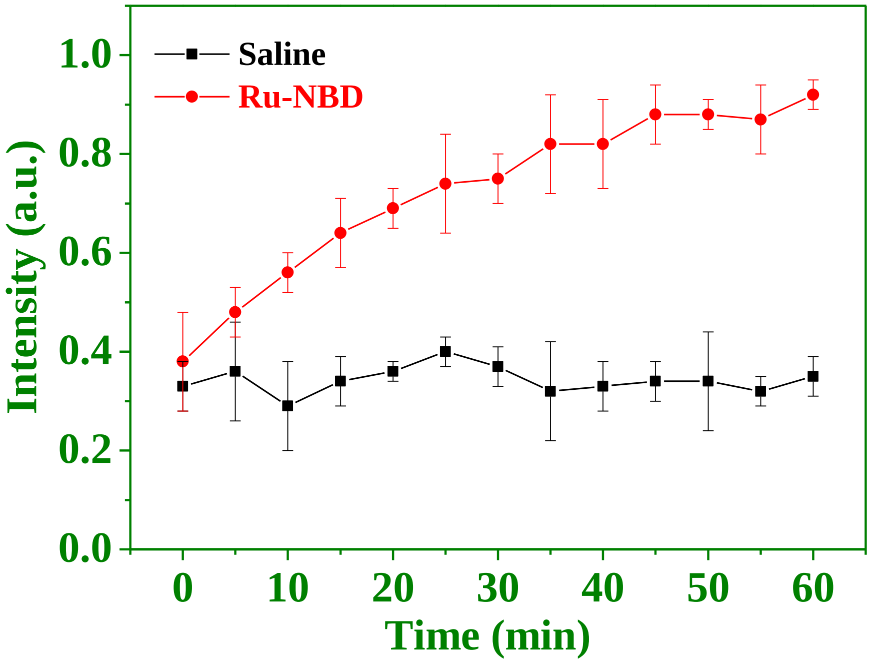

Figure S27. Luminescence imaging of biothiols in live mice using **Ru-NBD** as a probe. Ex: 465 nm, Em: 530 nm.

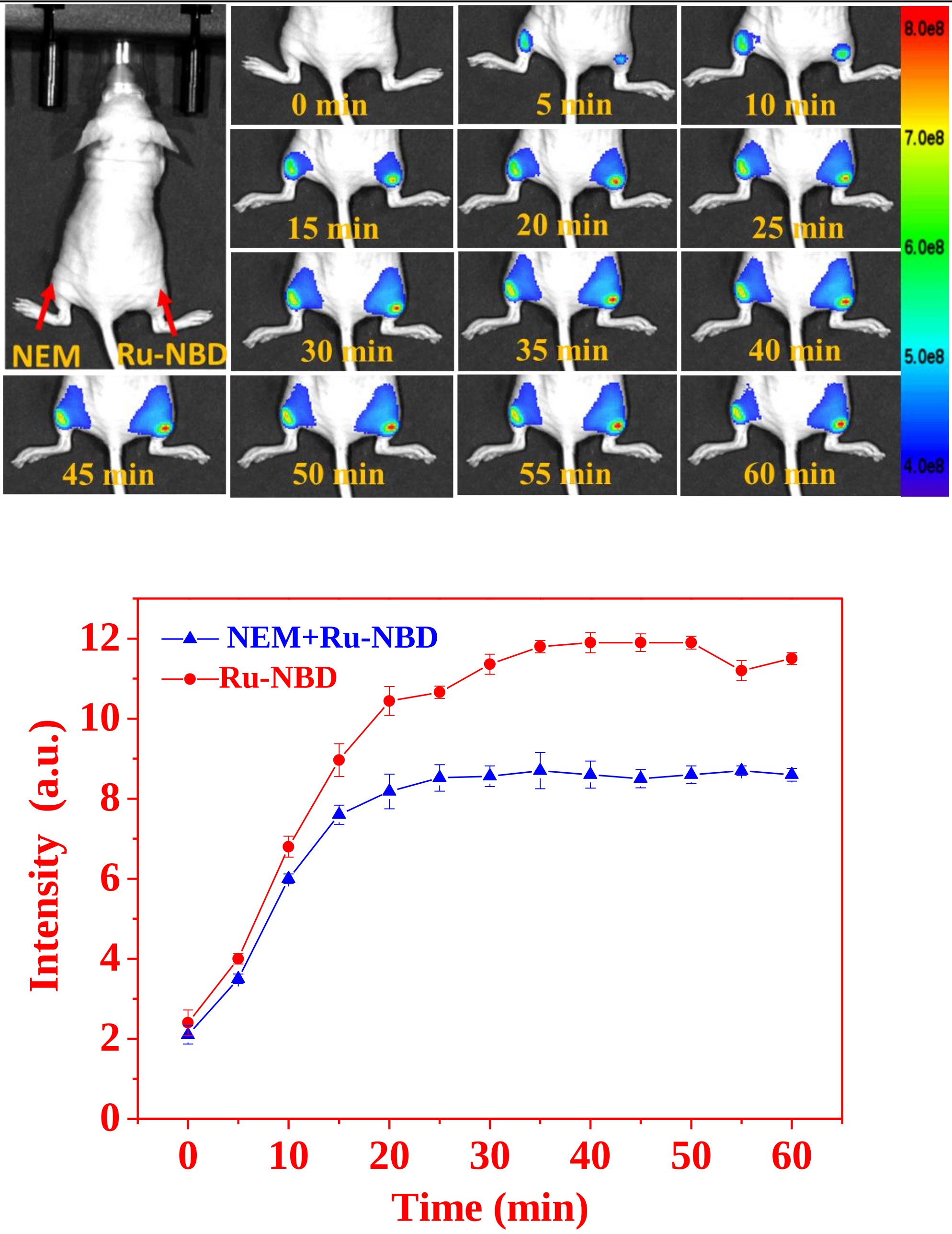

Figure S28. Luminescence imaging of biothiols in live mice using Ru-NBD as a probe. Ex: 465nm, Em: 610 nm.

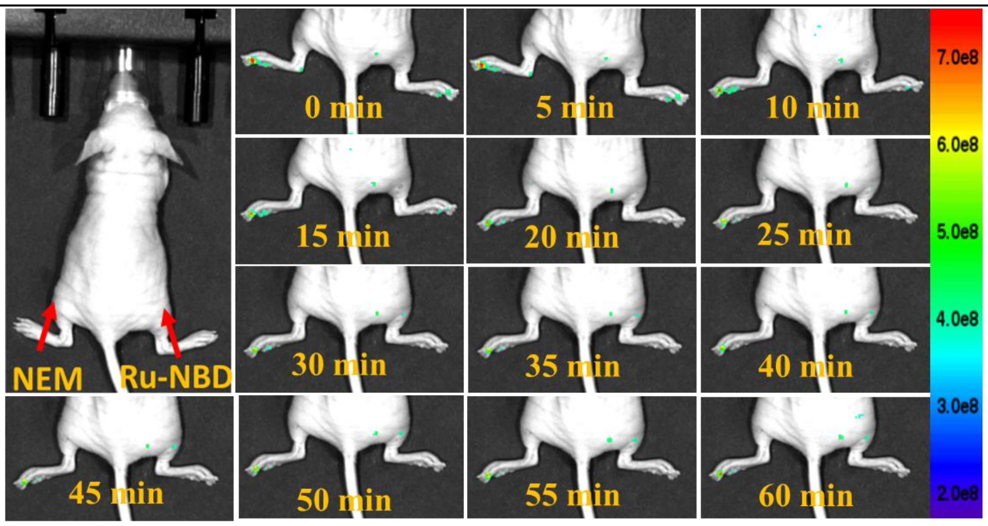

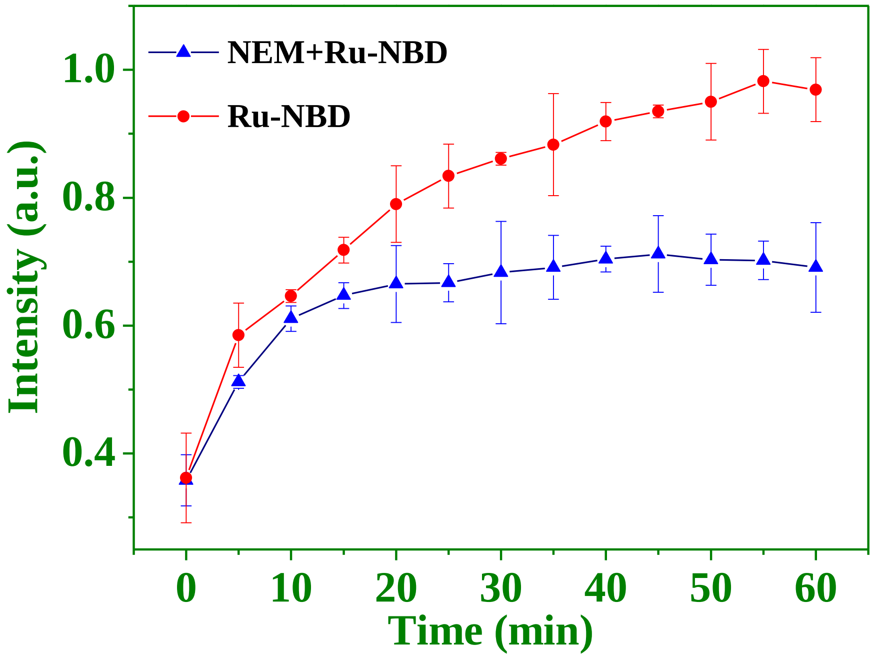

Figure S29. Luminescence imaging of biothiols in live mice using **Ru-NBD** as a probe. Ex: 465 nm, Em: 530 nm.

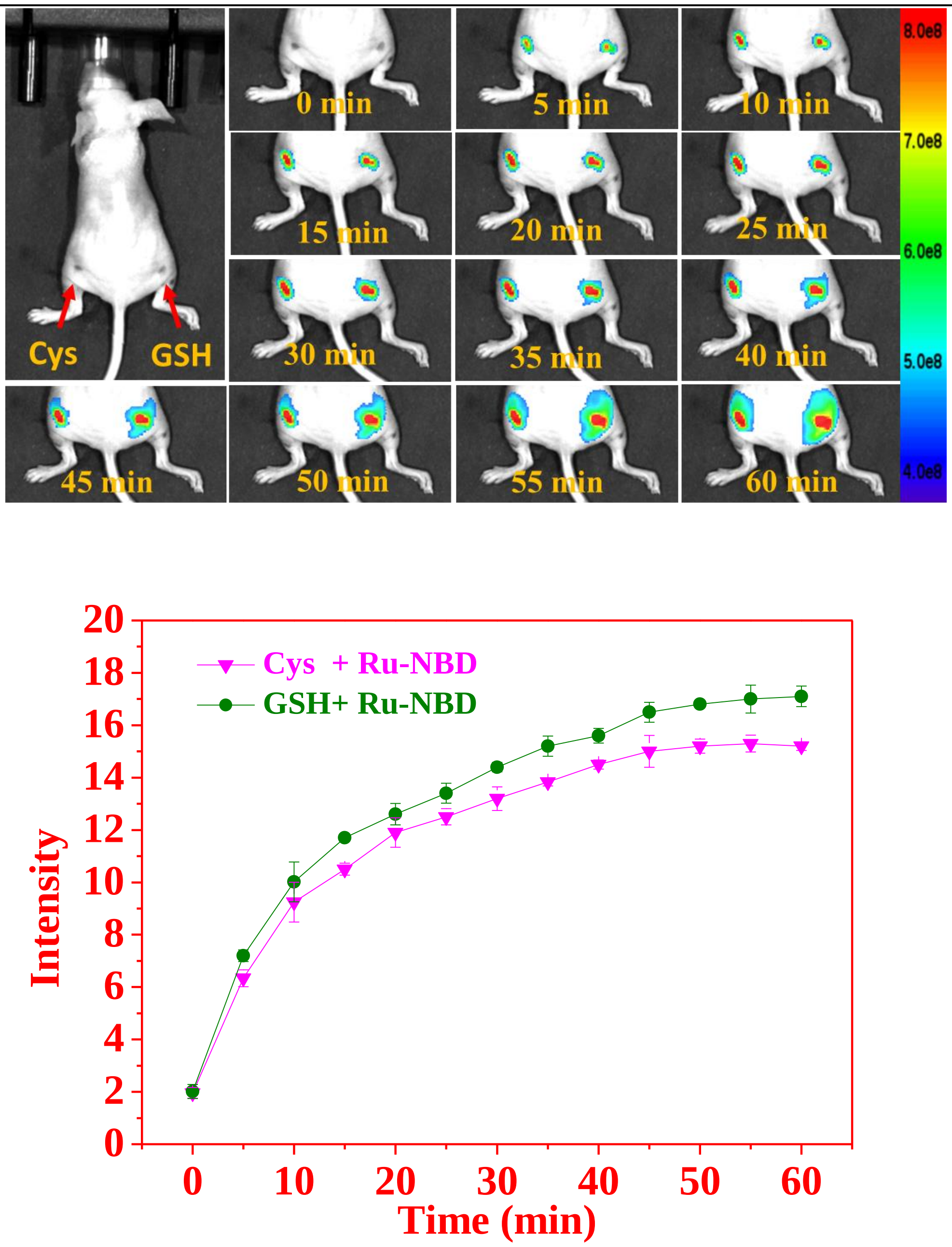

Figure S30. Luminescence imaging of biothiols in live mice using **Ru-NBD** as a probe. Ex: 465 nm, Em: 610 nm.

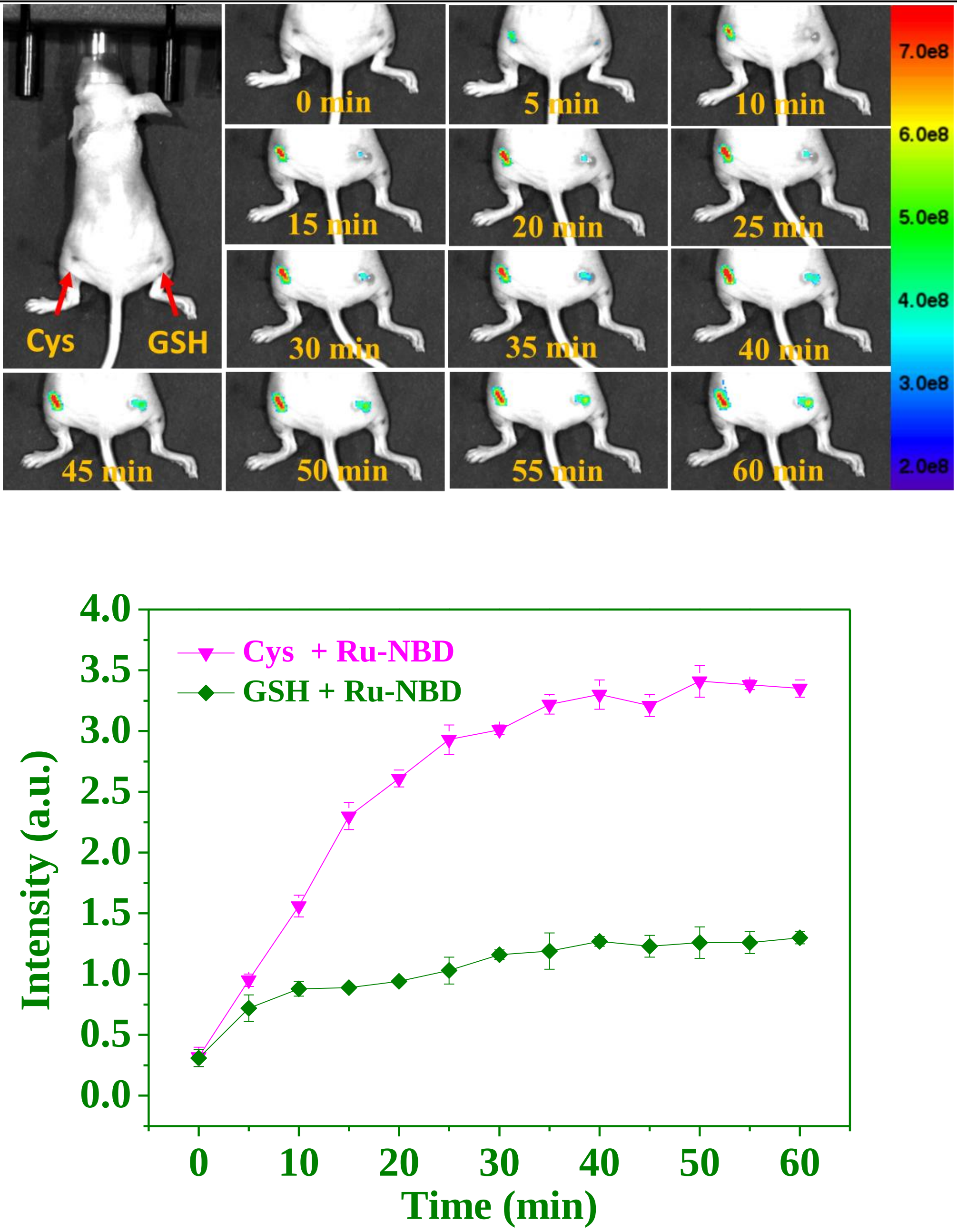

Figure S31. Luminescence imaging of biothiols in live mice using **Ru-NBD** as a probe. Ex: 465 nm, Em: 530 nm.

Table S6. Summary of the sensing performance of the probes in published papers and current work.

probe	λ_{ex}/λ_{em} (nm)	Detection limit （μM）	discrimination of biothiols	Detect total biothiols	Application	Literature
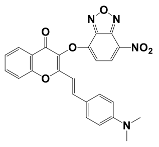	λ_{ex}: 458<br>λ_{em}: 621 (GSH)<br>621/545 (Cys/Hcy)	6.4 (GSH)<br>2.1 (Cys)<br>2.7 (Hcy)	discrimination GSH and Cys/Hcy	NO	cell	8
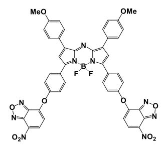	λ_{ex}: 470/670<br>λ_{em}: 730 (GSH)<br>540/730 (Cys/Hcy)	0.05 (GSH)<br>0.08 (Cys)<br>0.17 (Hcy)	discrimination GSH and Cys/Hcy	NO	cell	9
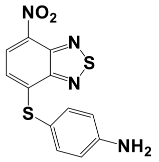	λ_{ex}: 475<br>λ_{em}: 535 (Cys/Hcy)	0.1 (Cys/Hcy)	discrimination Cys and Hcy	NO	cell	10
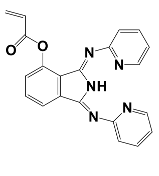	λ_{ex}: 368<br>λ_{em}: 585 (Cys/Hcy)	0.0054(Cys)<br>0.007 (Hcy)	NO	NO	cell	11
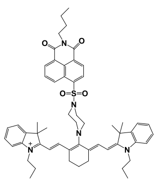	λ_{ex}: 370/700<br>λ_{em}: 495/795 (GSH)	0.153 (495)<br>0.171 (795)	NO	NO	Dual channel Cell	12
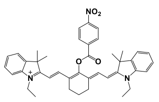	λ_{ex}: 580/720<br>λ_{em}: 640/785	0.2	NO	NO	ratio	13
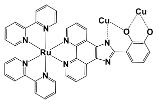	λ_{ex}: 465<br>λ_{em}: 585<br>(Cys)	1.1 (GSH)	NO	NO	Reversible Cell	14
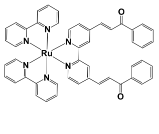	λ_{ex}: 470<br>λ_{em}: 620<br>(Cys)	3.2 (Cys)	NO	NO	Cell Zebrafish	15

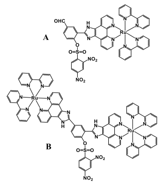	λ_{ex}: 461<br>λ_{em}: 608	A: 0.23 (GSH)<br>0.22 (Cys)<br>0.50 (Hcy)<br>B: 0.64 (GSH)<br>0.70 (Cys)<br>1.07 (Hcy)	NO	NO	Cell	16
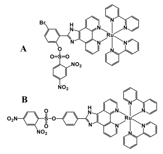	λ_{ex}: 458<br>λ_{em}: 608	A: 0.47 (Cys)<br>0.27 (GSH)<br>B: 0.30 (Cys)<br>0.24 (GSH)	NO	NO	Cell	17
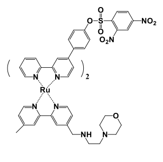	λ_{ex}: 459<br>λ_{em}: 620	0.062 (GSH)<br>0.146 (Cys)<br>0.115 (Hcy)	NO	Yes	Cell<br>Daphnia magna<br>TGL	18
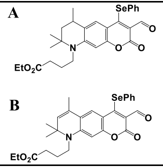	A: 526 (GSH)<br>390 (Cys/Hcy)<br>B: 550 (GSH)<br>410 (Cys/Hcy)	A: 0.31 (Cys)<br>0.01 (GSH)<br>B: 1.27 (Cys)<br>0.017 (GSH)	Discrimination of GSH and Cys/Hcy	Yes	Cell mice	19
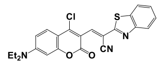	λ_{ex}: 480 (Cys)<br>360 (Hcy)<br>400 (GSH)<br>λ_{em}: 457(Cys)<br>559(Hcy) 529(GSH)	0.0005(Cys)<br>0.0036(Hcy)<br>0.0069(GSH)	Discrimination of GSH, Cys and Hcy	NO	Cell	20
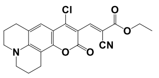	λ_{ex}: 375 (Hcy)<br>400 (Cys)<br>500 (GSH)<br>λ_{em}: 467(Hcy)<br>503(Cys) 568(GSH)	0.0007(Hcy)<br>0.0002(Cys)<br>0.001(GSH)	Discrimination of GSH, Cys and Hcy	NO	Cell	21
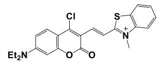	λ_{ex}: 360 (Cys)<br>450 (GSH)<br>λ_{em}: 420(Cys)<br>512(GSH)	0.05 (GSH)<br>＞0.4 (Cys)	Discrimination of GSH and Cys	NO	Cell	22
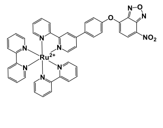	λ_{ex}: 480 (Cys)<br>λ_{em}:540 (Cys/Hcy)<br>628(GSH/Cys/Hcy)	0.1389(GSH)<br>0.1964(Cys)<br>0.0829(Hcy)	Discrimination of Cys/Hcy and GSH	Yes	Cell Zebrafish Mice TGL	This work

REFERENCE

[1] a) R. Zhang, Z. Ye, Y. Yin, G. Wang, D. Jin, J. Yuan, J. A. Piper, *Bioconjugate Chem.* **2012**, *23*, 725; b) Q. Gao, W. Zhang, B. Song, R. Zhang, W. Guo, J. Yuan, *Anal. Chem.* **2017**, *89*, 4517.

[2] M. J. Frisch, G. W. Trucks, H. B. Schlegel, G. E. Scuseria, M. A. Robb, J. R. Cheeseman, G. Scalmani, V. Barone, B. Mennucci, G. A. Petersson, H. Nakatsuji, M. Caricato, X. Li, H. P. Hratchian, A. F. Izmaylov, J. Bloino, G. Zheng, J. L. Sonnenberg, M. Hada, M. Ehara, K. Toyota, R. Fukuda, J. Hasegawa, M. Ishida, T. Nakajima, Y. Honda, O. Kitao, H. Nakai, T. Vreven, J. A. Montgomery Jr., J. E. Peralta, F. Ogliaro, M. J. Bearpark, J. Heyd, E. N. Brothers, K. N. Kudin, V. N. Staroverov, R. Kobayashi, J. Normand, K. Raghavachari, A. P. Rendell, J. C. Burant, S. S. Iyengar, J. Tomasi, M. Cossi, N. Rega, N. J. Millam, M. Klene, J. E. Knox, J. B. Cross, V. Bakken, C. Adamo, J. Jaramillo, R. Gomperts, R. E. Stratmann, O. Yazyev, A. J. Austin, R. Cammi, C. Pomelli, J. W. Ochterski, R. L. Martin, K. Morokuma, V. G. Zakrzewski, G. A. Voth, P. Salvador, J. J. Dannenberg, S. Dapprich, A. D. Daniels, Ö. Farkas, J. B. Foresman, J. V. Ortiz, J. Cioslowski, D. J. Fox, Gaussian, Inc., Wallingford, CT, USA **2009**.

[3] J. Wang, F. Q. Bai, B. H. Xia, H. X. Zhang, *J. Phys. Chem. A* **2011**, *115*, 11689.

[4] a) S. Ji, H. Guo, W. Wu, W. Wu, J. Zhao, *Angew. Chem. Int. Ed.* **2011**, *50*, 8283; b) K. Fujimoto, K. Kawai, S. Masuda, T. Mori, T. Aizawa, T. Inuzuka, T. Karatsu, M. Sakamoto, S. Yagai, T. Sengoku, M. Takahashi, H. Yoda, *Langmuir : the ACS journal of surfaces and colloids* **2019**, *35*, 9740.

[5] M. Cossi, V. Barone, *J. Chem. Phys.* **2001**, *115*, 4708.

[6] a) Y. Urano, M. Kamiya, K. Kanda, T. Ueno, K. Hirose, T. Nagano, *J. Am. Chem. Soc.* **2005**, *127*, 4888; b) K. Komatsu, K. Kikuchi, H. Kojima, Y. Urano, T. Nagano, *J. Am. Chem. Soc.* **2005**, *127*, 10197; c) X. Zhang, L. Chi, S. Ji, Y. Wu, P. Song, K. Han, H. Guo, T. D. James, J. Zhao, *J. Am. Chem. Soc.* **2009**, *131*, 17452.

[7] a) X. Yang, Y. Guo, R. M. Strongin, *Angew. Chem. Int. Ed.* **2011**, *50*, 10690; b) Y. Lyu, K. Pu, *Adv. Sci.* **2017**, *4*, 1600481; c) C. Liu, W. Chen, W. Shi, B. Peng, Y. Zhao, H. Ma, M. Xian, *J. Am. Chem. Soc.* **2014**, *136*, 7257.

[8] W. Chen, H. Luo, X. Liu, J. W. Foley, X. Song, *Anal. Chem.* **2016**, *88*, 3638.

[9] H. J. Xiang, H. P. Tham, M. D. Nguyen, S. Z. Fiona Phua, W. Q. Lim, J. G. Liu, Y. Zhao, *Chem. Commun.* **2017**, *53*, 5220.

[10] D. Lee, G. Kim, J. Yin, J. Yoon, *Chem. Commun.* **2015**, *51*, 6518.

[11] X. Liu, H. Tian, L. Yang, Y. Su, M. Guo, X. Song, *Tetrahedron Lett.* **2017**, *58*, 3209.

[12] Z. Xu, X. Huang, X. Han, D. Wu, B. Zhang, Y. Tan, M. Cao, S. H. Liu, J. Yin, J. Yoon, *Chem* **2018**, *4*, 1609.

[13] K. Yin, F. Yu, W. Zhang, L. Chen, *Biosens. Bioelectron.* **2015**, *74*, 156.

[14] P. Zhang, Y. Wang, W. Huang, Z. Zhao, H. Li, H. Wang, C. He, J. Liu, Q. Zhang, *Sens. Actuators, B* **2018**, *255*, 283.

[15] Y. Li, N. Shi, M. Li, *New J. Chem.* **2019**, *43*, 18517.

[16] Z. B. Zheng, Y. F. Han, Y. Q. Ge, J. C. Cui, J. Zuo, K. Nie, *Spectrochim. Acta, Part A* **2019**, *216*, 328.

[17] Z.-B. Zheng, J.-C. Cui, Y.-F. Han, Y.-Q. Ge, J. Zuo, W.-X. Hao, *Anal. Methods* **2019**, *11*, 2341.

[18] Q. Gao, W. Zhang, B. Song, R. Zhang, W. Guo, J. Yuan, *Anal. Chem.* **2017**, *89*, 4517.

[19] S. V. Mulay, Y. Kim, M. Choi, D. Y. Lee, J. Choi, Y. Lee, S. Jon, D. G. Churchill, *Anal. Chem.* **2018**, *90*, 2648.

[20] G. X. Yin, T. T. Niu, Y. B. Gan, T. Yu, P. Yin, H. M. Chen, Y. Y. Zhang, H. T. Li, S. Z. Yao, *Angew. Chem., Int. Ed. Engl.* **2018**, *57*, 4991.

[21] G. Yin, T. Niu, T. Yu, Y. Gan, X. Sun, P. Yin, H. Chen, Y. Zhang, H. Li, S. Yao, *Angew. Chem., Int. Ed. Engl.* **2019**, *58*, 4557.

[22] J. Liu, Y. Q. Sun, Y. Huo, H. Zhang, L. Wang, P. Zhang, D. Song, Y. Shi, W. Guo, *J. Am. Chem. Soc.* **2014**, *136*, 574.
